# Application of Biotechnology in the Synthesis of Nanoparticles—A Review

**DOI:** 10.3390/molecules31091415

**Published:** 2026-04-24

**Authors:** Abayomi Baruwa, Oluwatoyin Joseph Gbadeyan, Kugenthiren Permaul

**Affiliations:** 1 Department of Biotechnology and Food Science, Durban University of Technology, Durban 4000, South Africa; abayomi.baruwa@gmail.com (A.B.); kugen@dut.ac.za (K.P.); 2 Institute of System Science, Durban University of Technology, Durban 4000, South Africa

**Keywords:** nanoparticles, nanotechnology, green synthesis, biotechnological applications

## Abstract

The field of nanoparticle-based biotechnology has undergone substantial advancement, characterized by progress in targeted drug delivery systems, the development of innovative diagnostic and imaging platforms, the expanded adoption of environmentally sustainable (“green”) synthesis approaches, and an increasing emphasis on the integration of emerging technologies such as artificial intelligence and nanorobotics. Conventional nanoparticle synthesis often involves toxic reducing agents; however, recent advances promote eco-friendly green synthesis methods utilizing biological systems such as bacteria, fungi, algae, yeast, plants, and actinomycetes. These biological approaches are safe, sustainable, cost-effective, and capable of producing highly stable Nanoparticles (NPs). The interaction of nanomaterials with biological systems is crucial for developing intracellular and subcellular drug delivery technologies with minimal toxicity, governed by nano–bio interface mechanisms such as cellular translocation, surface wrapping, embedding, and internal attachment. Key factors influencing NP behavior include morphology, size, surface area, surface charge, and ligand chemistry. Magnetic nanoparticles, particularly iron-based forms, exhibit unique superparamagnetic properties that are strongly influenced by particle size, as explained by the Néel relaxation mechanism, in which thermal energy induces flipping of magnetic moments. Nanoparticles demonstrate diverse modes of action, including antimicrobial activity, reactive oxygen species (ROS)-induced cytotoxicity, genotoxicity, and plant growth promotion. NP performance and biological effects are strongly dependent on their size, shape, dosage, and concentration. This critical review article aims to elucidate evolution, classification, preparation methods, and multifaceted applications of nanoparticles.

## 1. Introduction

A nanoparticle is a discrete particle of matter with at least one external dimension in the size range of approximately 1 to 100 nanometres (nm), in which size-dependent physicochemical properties, such as increased surface area-to-volume ratio, quantum effects, and altered reactivity, distinguish it from its bulk counterpart [1].

Nanoparticles may be composed of organic, inorganic, or hybrid materials and can exist in various morphologies (e.g., spherical, rod-shaped, or irregular). Their nanoscale dimensions confer unique optical, electrical, mechanical, and biological properties, making them particularly valuable for applications in biotechnology, medicine, and materials science [2].

Nanoparticles (NPs) are attracting great interest in technology because they can be easily tailored and often work more efficiently than larger-sized materials [3]. Traditionally, nanoparticles (NPs) are produced by reducing metal ions into nanosized particles using chemical reducing agents, which are often harmful. Recently, researchers have been focusing on eco-friendly methods that use natural resources to make nanoparticles instead [3]. Green synthesis is a new approach that uses biological methods to produce nanoparticles (NPs). It is gaining attention for being environmentally friendly, safe, low-cost, simple, and highly efficient [4].

Different biological systems, such as bacteria, actinomycetes, fungi, algae, yeast, and plants, can serve as natural factories for producing nanoparticles, with great potential to advance sustainable nanotechnology [4]. Nanoparticles (NPs) are widely used in many areas, including environmental science, agriculture, food technology, biotechnology, biomedicine, and pharmaceuticals [5]. Specific uses include wastewater treatment, environmental monitoring, functional food additives, and antimicrobial agents [5].

Nanoparticles (NPs) have special properties, such as biocompatibility, anti-inflammatory and antibacterial effects, effective drug delivery, and the ability to target tumors. They also improve bioavailability, bioactivity, and absorption. These features have greatly expanded their use in biotechnology and applied microbiology [5]. The unique properties of nanoparticles (NPs) come from their very small size and extremely large surface area [6]. When particle size becomes as small as, or smaller than, the de Broglie wavelength or the wavelength of light, the regular arrangement of their crystal structure is disturbed [6].

This effect endows nanoparticles with physical and chemical properties that differ significantly from those of larger materials, enabling new and diverse applications across many fields [6]. Nanoparticles (NPs) are usually between 1 and 100 nanometers in size [7]. Nanoparticles can be produced in two main ways: the top-down and bottom-up methods [7]. The science of nanoparticles relies primarily on two aspects: achieving well-controlled size and shape, and exploring specific applications [8]. Controlled size synthesis of nanoparticles is achieved using different stabilizing agents such as ligands, surfactants, and polymers [5].

**Figure 1:**
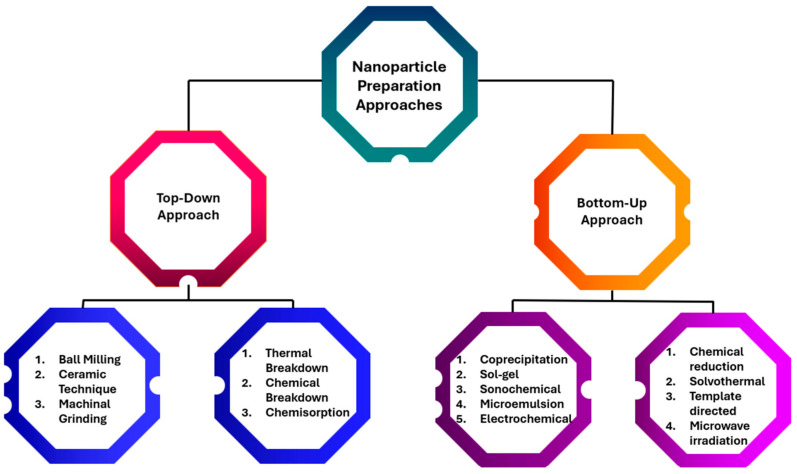
Summary of nanoparticle preparation methods.

The selectivity and reactivity of nanoparticles are crucial factors influenced by their surface area [9]. Carefully controlling the surface structure, shape, and composition of nanoparticles is essential because these factors determine their selectivity and reactivity [10]. One ongoing challenge in the industrial use of nanocatalysts is recyclability. Magnetic nanoparticles have proven effective for separation, offering benefits over traditional methods such as fluid extraction, chromatography, and filtration [10]. Nanocatalysts attached to superparamagnetic nanomaterials can be easily removed using an external magnetic field. This allows them to be reused without needing the magnetic field [10]. A summary of nanoparticle preparation methods is elucidated in Figure 1.

The primary aim of this work is to provide a comprehensive and critically informed overview of the progression, design, and biotechnological applications of nanoparticles, with particular emphasis on their evolving roles as functional nanocarriers in areas such as drug delivery, phytomedicine, diagnostics, and environmental biotechnology. The study seeks to elucidate how advances in nanoparticle engineering, including size control, surface functionalization, and biocompatibility, have expanded their applicability, while also addressing key limitations related to toxicity, stability, and translational feasibility. A specific objective is to highlight emerging nanostructures, such as dendrimers and lipid-based systems, and to evaluate their potential and constraints within complex biological environments.

To facilitate a clear and logical flow of ideas, this article is structured as follows. The introduction outlines the fundamental concepts and historical development of nanoparticles in biotechnology. This is followed by a section detailing the classification and synthesis strategies of nanoparticles, emphasizing physicochemical properties relevant to biological interactions. Subsequent sections explore major applications, including drug delivery systems, phytochemical encapsulation, diagnostic imaging, and environmental remediation. A dedicated section critically examines the challenges associated with nanoparticle use, particularly issues of cytotoxicity, bioaccumulation, and regulatory considerations. The review then discusses recent innovations and future perspectives aimed at overcoming these barriers. Finally, the conclusion synthesizes key insights and underscores the potential of nanoparticles to drive transformative advances in biotechnology.

Methodology for Literature Selection

A systematic and reproducible literature search strategy was employed to compile and analyze current advances in the application of biotechnology in nanoparticle synthesis. Multiple electronic databases were consulted to ensure comprehensive coverage of relevant peer-reviewed studies. These included PubMed, Scopus, Web of Science, and Google Scholar, selected for their extensive indexing of biomedical, biochemical, and nanotechnology research.

The search was conducted using well-defined keywords and Boolean combinations to maximize retrieval efficiency and relevance. Core search terms included: “biogenic nanoparticle synthesis”, “green synthesis of nanoparticles”, “microbial nanoparticle synthesis”, “plant-mediated nanoparticles”, “fungal synthesis of nanoparticles”, “enzyme-assisted nanoparticle synthesis”, “nanobiotechnology”, and “biosynthesis of metal nanoparticles”. These terms were combined using operators such as AND, OR, and NOT to refine results (e.g., “green synthesis AND nanoparticles AND biotechnology” or “microbial synthesis OR plant-mediated nanoparticles”).

To ensure the inclusion of high-quality and relevant studies, predefined inclusion and exclusion criteria were applied. Inclusion criteria comprised: (i) peer-reviewed research articles and review papers; (ii) studies focusing on biological or biotechnological methods for nanoparticle synthesis; (iii) publications written in English; and (iv) studies published within the last 10–15 years to capture recent advancements. Exclusion criteria included: (i) duplicate records; (ii) studies lacking experimental or methodological clarity; (iii) articles not directly related to nanoparticle synthesis via biological systems; and (iv) conference abstracts, editorials, and non-peer-reviewed sources.

The selection process followed a stepwise screening approach. Initially, titles and abstracts were evaluated for relevance, followed by full-text assessment of shortlisted articles. Emphasis was placed on studies detailing synthesis mechanisms, characterization techniques, scalability, and applications in biotechnology. This structured approach ensured that the final selection of literature was both comprehensive and aligned with the objectives of the review.

### 1.1. Evolution of Nanotechnology

Nanotechnology emerged as a transformative field in the 1980s, driven by pivotal experimental breakthroughs [3]. The invention of the scanning tunneling microscope in 1981 made it possible to see and control matter at the atomic level, while the discovery of fullerenes in 1985 provided a deeper understanding of nanoscale structures [11]. Nanotechnology gained widespread attention in 1986 with the publication of Engines of Creation, which played a key role in popularizing the potential applications and impact of nanotechnology [4].

### 1.2. Inception of NPs

Carbon nanotubes have been discovered in pottery from Keeladi, India, dating back to around 600–300 BCE [3]. Cementite nanowires have also been found in Damascus steel, a famous material that dates back to around 900 CE [3]. Despite these findings, the processes leading to the formation of these nanostructures remain unclear, and it is uncertain whether their presence in these materials was intentional or incidental [5].

### 1.3. Detection of C, Ag, Zn, Cu, and Au Nanoparticles

The identification of carbon nanoparticles (NPs) in 1991 was a key milestone in nanotechnology, soon followed in 1993 by Iijima and Ichihashi’s synthesis of single-walled carbon nanotubes (SWCNTs), with diameters of approximately 1 nanometer [3,12]. Carbon nanotubes (CNTs), also called Bucky tubes, are nanomaterials made by rolling a sheet of carbon atoms arranged in a hexagonal pattern into a hollow cylinder [6]. As carbon allotropes, CNTs bridge the gap between fullerenes (0-dimensional structures) and graphene (2-dimensional sheets) [5]. Nearly 120 years ago, M.C. Lea reported the synthesis of citrate-stabilized silver colloids, creating particles about 7–9 nm in size [13]. This method is analogous to modern nanosilver synthesis, which uses silver nitrate and citrate as stabilizing agents [13]. Proteins were also documented as stabilizers for nanosilver as early as 1902 [3]. Commercial production of nanosilver, known as “Collargol,” began in 1897, with particle sizes around 10 nm determined by 1907 [3]. In 1953, Moudry developed gelatin-stabilized silver nanoparticles, sized between 2 and 20 nm, using a method different from Collargol [13]. Early patents emphasized the importance of nanoscale silver, specifying that colloidal particles below 25 nm offered optimal efficiency [4,11]. Gold nanoparticles (AuNPs) have a long history, dating back to the Roman era, when they were used to stain glassware. The modern study of gold nanoparticles (AuNPs) began in 1857, when Michael Faraday examined colloidal “ruby” gold and discovered its unique optical and electrical properties [6]. Faraday showed that these nanoparticles could form solutions of different colors when exposed to specific lighting conditions, distinguishing their behavior from that of bulk gold [6]. Gold nanoparticles (AuNPs) remain essential in nanotechnology due to their unique properties and wide range of applications [5].

### 1.4. Categories of NPs

Nanoparticles (NPs) are grouped into categories based on shape, size, and chemical properties, which determine their unique physical, chemical, and functional characteristics. These classifications enable their tailored application in various scientific and industrial fields.

#### 1.4.1. Carbonaceous Nanoparticles

Fullerenes and carbon nanotubes (CNTs) are two main types of carbon-based nanoparticles (NPs) [3]. Fullerenes are spherical, hollow cage-like structures made of sp^2^-hybridized carbon atoms arranged in pentagons and hexagons [3]. These carbon allotropes have exceptional properties, including high electrical conductivity, great mechanical strength, strong electron affinity, and flexible structures, which have attracted major scientific and economic interest [11]. In contrast, CNTs are elongated nanostructures resembling rolled-up sheets of graphene, with diameters typically ranging from 1–2 nm [11]. Carbon nanotubes (CNTs) are classified based on the number of concentric graphene layers: single-walled (SWCNTs), double-walled (DWCNTs), or multi-walled (MWCNTs). Their tubular structure gives them outstanding mechanical, thermal, and electrical properties, making them highly useful in advanced technologies [11].

#### 1.4.2. Metal NPs

Metal nanoparticles (NPs) are made entirely of metals and are known for their unique electrical properties, which mainly come from a phenomenon called localized surface plasmon resonance (LSPR) [3]. Metals like copper (Cu), silver (Ag), and gold (Au) exhibit broad visible-range absorption bands, which improve their optical performance [5]. These nanoparticles are widely used across many scientific fields because their facets, sizes, and shapes can be precisely controlled during synthesis, endowing them with advanced properties [5]. Because their properties can be tuned, metal nanoparticles (NPs) are highly valuable for applications in photonics, catalysis, biomedicine, and environmental science [6].

#### 1.4.3. Oxide Nanoparticles

Over the past decade, metal oxide nanoparticles (MONPs) have attracted considerable attention across interdisciplinary fields due to their unique physicochemical properties, which differ markedly from their bulk counterparts [14]. As particle size decreases, the surface-to-volume ratio increases, allowing surface atoms to dominate overall material behavior, thereby altering optical, electronic, and catalytic properties [13]. These tunable characteristics have enabled the fabrication of advanced nanostructures for diverse industrial and biomedical applications, including catalysis, energy conversion, electronics, and medicine [11]. Specifically, magnesium oxide (MgO) nanoparticles stand out for their wide band gap, chemical stability, non-toxicity, and environmental compatibility, making them suitable for use in biomedicine and biotechnology [15]. MgO nanostructures can be synthesized through chemical (bottom-up) or physical (top-down) methods, with their morphology and properties controlled by reaction parameters and precursor composition [11].

#### 1.4.4. Lipid-Based NPs

Lipid nanoparticles (NPs) are commonly used in biological applications because they are made of lipid components. They are spherical and usually range in size from 10 to 1000 nm. Structurally, they have a solid lipid core surrounded by soluble lipophilic molecules, which makes them especially useful for drug delivery, gene therapy, and other biomedical applications. Lipid nanoparticles can reach sizes of up to 1000 nm because their dimensions are tailored to meet the requirements of different drug-delivery and cosmetic applications. Their size influences key properties, such as surface area, cellular uptake, stability, and drug release rate. Within this size range, they can efficiently carry both hydrophilic and lipophilic drugs, protecting them from degradation and enabling controlled or sustained release. The particle size can be precisely adjusted during production using specific formulation and processing techniques to achieve different functional goals, such as enhancing skin penetration or targeting particular tissues or cells [4].

##### Liposomes as the Most Actively Used Nanoparticles

Liposomes are tiny spherical vesicles used to deliver a wide range of therapeutic agents. They are versatile because they can carry both water-soluble and fat-soluble drugs, making them especially useful for combination therapies [16]. Liposomes have a long history in medicine, with established formulations for treating cancer and fungal infections [16]. However, liposomes can leak or degrade during storage, and their circulation time in the body is often limited unless they are specially modified [17]. Producing liposomes on a large scale is also challenging because achieving uniform size and consistent drug loading is technically difficult [18]. In cancer therapy, liposomal drugs like doxorubicin (e.g., Doxil^®^) improve drug delivery to tumors by taking advantage of the enhanced permeability and retention (EPR) effect, while reducing harmful effects on the heart [15]. In antifungal treatments, liposomes such as Ambisome^®^ deliver amphotericin B more safely, lowering toxicity [15]. Liposomes are also used in vaccines to deliver antigens and adjuvants, thereby strengthening immune responses. Additionally, they are being explored as carriers for monoclonal antibodies, providing targeted therapies for autoimmune diseases and certain cancers [19].

##### Comparison Between Lipid Nanoparticles (LNPs) and Liposomes

Lipid nanoparticles (LNPs) are an advanced type of lipid-based delivery system, like liposomes, but with important differences in structure, production, and use [19]. Liposomes are vesicles that have a water-filled core, whereas LNPs, including solid lipid nanoparticles (SLNs), are made of a solid lipid matrix rather than a liquid core [2]. Although both are lipid-based and share some functional properties, their structural differences make LNPs better suited for certain pharmaceutical applications, such as delivering nucleic acids, poorly soluble drugs, or providing enhanced stability [20]. Lipid nanoparticles (LNPs) represent a modern evolution of lipid-based drug delivery systems, building on traditional liposome technology to provide enhanced stability, higher drug-loading capacity, and more efficient delivery of complex biologics such as mRNA and siRNA [1]. One key advantage of LNPs is their scalability; production methods for LNPs, including solid lipid nanoparticles (SLNs) and nanostructured lipid carriers (NLCs), are more readily adapted to industrial-scale manufacturing than conventional liposome techniques [1]. Both liposomes and LNPs remain important in 2025; liposomes continue to dominate the market for well-established therapeutic products, while LNPs are leading the rapidly expanding fields of genetic medicine and next-generation vaccines [21]. The market for both delivery systems is experiencing strong growth and ongoing clinical development, often utilizing advanced technologies such as artificial intelligence to optimize formulations and improve therapeutic outcomes [21].

Structure

Liposomes are spherical vesicles made of one or more lipid bilayers that enclose a water-filled core. In contrast, lipid nanoparticles (LNPs) consist of a solid or semi-solid lipid core surrounded by a single phospholipid layer, providing structural stability and efficient drug delivery [1].

Production Technology

Liposomes are produced using complex methods such as mechanical dispersion, solvent injection, and microfluidization, which can make large-scale production challenging. In contrast, lipid nanoparticles (LNPs) are manufactured using advanced, more easily scalable techniques, often high-energy methods like microfluidics, that allow better control over particle size and consistency for industrial production [22].

Stability and size

Liposomes: Liposomes are relatively large, typically 50–1000 nm. Their larger size allows them to carry more therapeutic agents but also makes them more prone to leakage and degradation during storage [22]. To improve stability and extend circulation time in the body, liposomes are often coated with polyethene glycol (PEG) or other stabilizing agents. In contrast, LNPs are smaller, usually between 20 and 100 nanometers, which enhances their stability during storage [23]. Their compact structure reduces the risk of drug leakage, aggregation, or degradation, making them especially suitable for sensitive therapies such as genetic treatments, where preserving the integrity of the payload is critical [23].

Drug Loading

Liposomes can carry both water-soluble (hydrophilic) drugs in their aqueous core and fat-soluble (hydrophobic) drugs within their lipid bilayer. In contrast, lipid nanoparticles (LNPs) offer higher drug-loading capacity and improved bioavailability, making them especially effective for delivering lipophilic drugs and large molecules like nucleic acids [23].

Commercial Availability & Application

Liposomes are a well-established technology with over 50 years of use and several approved products, such as Doxil^®^ for cancer therapy [24]. They are commonly applied in cancer treatment, antifungal therapies, and protein delivery. In contrast, Lipid Nanoparticles (LNPs) represent a rapidly growing field, propelled by the success of the Pfizer-BioNTech and Moderna mRNA COVID-19 vaccines [24]. LNPs are widely used in gene therapy, immunotherapy, and ongoing clinical trials for cancer and other advanced treatments [14].

Structural and Compositional Differences

Liposomes are spherical vesicles made of one or more lipid bilayers surrounding a water-filled core. This structure allows them to carry both water-soluble (hydrophilic) drugs in the core and fat-soluble (hydrophobic) drugs within the bilayer, making them versatile for a wide range of therapies [14]. Liposomes are mainly composed of phospholipids and cholesterol, which provide stability and controlled drug release. However, they are generally less efficient at delivering drugs directly into cells, favoring extracellular or gradual release instead. In contrast, Lipid nanoparticles (LNPs) are solid or semi-solid lipid particles stabilized by surfactants or other agents [25]. Their hydrophobic lipid core is ideal for encapsulating genetic materials, such as messenger RNA (mRNA) or small interfering RNA (siRNA). LNPs typically include ionizable lipids, cholesterol, phospholipids, and polyethylene glycol (PEG), which together promote efficient endosomal escape, allowing the therapeutic payload to reach intracellular targets [26]. LNPs have been essential for mRNA-based vaccines, such as Pfizer-BioNTech and Moderna COVID-19 vaccines, protecting fragile mRNA and ensuring its delivery to cells to trigger immune responses. While highly effective, LNPs can sometimes trigger immune reactions, and challenges remain in ensuring consistent manufacturing and controlled intracellular drug release [26]. Beyond vaccines, LNPs are being explored for gene-silencing therapies using siRNA, delivery of CRISPR-Cas9 for gene editing, and cancer immunotherapy by transporting tumor antigens or immunostimulatory agents to boost anti-cancer immunity [14].

#### 1.4.5. Semiconductor Nanocrystals

Semiconductor nanoparticles (NPs) have properties of both metals and non-metals, giving them unique physical and chemical characteristics. For example, they can absorb and emit light, which makes them useful in applications such as high-efficiency solar cells and brighter light-emitting diodes (LEDs) [4]. Their small size and improved properties also make it possible to create faster and more compact electronic devices, such as transistors [6]. Semiconductor nanoparticles (NPs) are also promising for biomedical uses, such as bio-imaging and cancer therapy, because they can interact with biological systems in unique ways [6].

#### 1.4.6. Polymer-Based Nanoparticles

Polymeric nanoparticles (NPs), usually 1–1000 nm in size, can carry active substances either by attaching them to the surface of the polymer core or by encapsulating them inside the polymer matrix [6]. These nanoparticles are mainly organic and are often referred to as polymer nanoparticles (PNPs) in the scientific literature. They usually appear as nanospheres or nanocapsules, depending on their structure and how they are made [6].

### 1.5. Categories of Metal Nanoparticles

Metal nanoparticles (NPs) are made entirely from metals and have unique optical and electrical properties, mainly caused by a phenomenon called localized surface plasmon resonance (LSPR) [5]. Nanoparticles made from alkali and noble metals, like copper (Cu), silver (Ag), and gold (Au), show broad absorption bands in the visible part of the solar spectrum [5]. Carefully controlling the synthesis of metal nanoparticles (NPs), including their facets, size, and shape, is essential for creating advanced materials with specific properties for high-tech applications [12].

#### 1.5.1. Silver Nanoparticles (AgNPs)

Silver nanoparticles (AgNPs) are tiny particles of silver, ranging in size from 1 to 100 nanometers [12]. Silver nanoparticles (AgNPs) exhibit unique physical and chemical properties due to their small size, large surface area, and ability to absorb and scatter light in the visible and near-infrared regions [11]. These features make silver nanoparticles (AgNPs) behave differently from bulk silver, giving them stronger antimicrobial properties than ionic silver [4]. Silver nanoparticles (AgNPs) can be produced in various sizes and shapes, depending on the production method, with chemical reduction being the most commonly used approach [4]. In a typical synthesis, silver nanoparticles (AgNPs) are made by chemically reducing a 12 mM aqueous solution of silver nitrate (AgNO_3_). The reaction is carried out under an argon atmosphere using 70 mL of a solution containing polyvinylpyrrolidone (PVP) at a PVP-to-silver molar ratio of 34:1, along with 21 mL of Aloe Vera extract [4]. The mixture is first sonicated for 45 min at room temperature, then heated at 2 °C/min to 80 °C and held at that temperature for 2 h. The solution becomes clear when small suspended particles are removed by simple filtration [4].

#### 1.5.2. Zinc-Based Nanoparticles

Zinc oxide nanoparticles (ZnNPs) are tiny particles made of zinc, with sizes between 1 and 100 nm. Zinc is a wide-band-gap semiconductor with an energy gap of 3.37 eV at room temperature. Because of their catalytic, electrical, optoelectronic, and photochemical properties, ZnNPs have attracted significant interest for many applications, especially in catalysis [5]. Zinc nanoparticles can be synthesized using various methods, such as laser ablation, hydrothermal techniques, electrochemical deposition, sol–gel processes, chemical vapor deposition, thermal decomposition, combustion methods, ultrasound- or microwave-assisted synthesis, mechanochemical-thermal methods, anodization, co-precipitation, electrophoretic deposition, and precipitation processes [5]. These methods enable precise control over the production of zinc nanoparticles, thereby tailoring their properties for different applications. Although Zinc oxide (ZnO) nanoparticles are considered to have limited biocompatibility due to their ability to release zinc ions and generate reactive oxygen species (ROS), which can induce oxidative stress and result in cellular damage or death [2]. The primary mechanism of toxicity involves the dissolution of ZnO nanoparticles within cells, releasing Zn^2+^ ions that interfere with essential cellular processes such as enzyme function and protein metabolism. In addition, ZnO nanoparticles can stimulate excessive ROS production, leading to oxidative stress, DNA damage, and apoptosis (programmed cell death). They may also directly disrupt cell membranes through physical interactions, leading to the leakage of intracellular components. Several factors influence the extent of ZnO nanoparticle toxicity, including particle size (with smaller particles exhibiting greater reactivity), surface modifications (which can reduce or alter toxicity), cell type (as some cells, including cancer cells, are more sensitive to ROS), and dosage (toxicity increases in a dose-dependent manner) [24]. Although these mechanisms raise concerns about biocompatibility, toxicity remains a complex issue, and ongoing research aims to develop surface-engineered ZnO nanoparticles that are safer and better suited for biomedical applications such as drug delivery and medical imaging [27].

#### 1.5.3. Copper Nanoparticles (CuNPs)

Copper nanoparticles (CuNPs) are tiny particles made of copper, with sizes between 1 and 100 nanometers [3]. The fluorescence properties of both copper (Cu) and gold (Au) metals have been well studied and documented [3]. When excited at 488 nm, a fluorescence peak appears near the interband absorption edge of the metals [5]. Interestingly, fluorescence peaks appear at the same energy for two different excitation ranges (457.9–514.5 nm and 300–400 nm), while the high-energy side of the peak becomes stronger as the photon energy increases [5]. A unique top-down method called Electrothermal Explosion Welding (EEW) has been used to produce copper nanoparticles (CuNPs) [13]. In this method, a current of 1010 A/m^2^ is passed through a thin copper wire, causing it to explode onto a copper plate in just 10^−6^ s, which produces copper nanoparticles [13].

#### 1.5.4. Gold-Based Nanoparticles

Gold nanoparticles (AuNPs) are tiny particles made of gold that have unique physical and chemical properties. They can absorb and scatter light in the visible and near-infrared ranges, making them useful for many applications [3]. Gold nanoparticles can form conjugates with PAMAM dendrimers, and such complexes exhibit potential applicability in diagnostic applications [1]. Around the beginning of the 20th century, scientists discovered anisotropic gold nanoparticles [3]. In his 1909 book, Zsigmondy noted that gold particles smaller than 40 nm are not always spherical [11]. He also identified anisotropic gold particles that exhibited a variety of colors [11]. Zsigmondy was awarded the Nobel Prize in 1925 for his pioneering work on the heterogeneous nature of colloidal solutions and for developing the ultramicroscope, which allowed him to see the shapes of gold particles [3]. He observed that gold particles often formed six-sided, leaf-like crystals. Because of their unique optical, electrical, and molecular recognition properties, gold nanoparticles (AuNPs) have been widely studied. They have great potential for applications in areas such as electron microscopy, electronics, nanotechnology, materials science, and biomedicine [3].

#### 1.5.5. Aluminum-Derived Nanoparticles

Aluminum nanoparticles (AlNPs) are tiny particles made entirely of aluminum at the nanometer scale [3]. Because of their high reactivity, AlNPs are useful for high-energy materials, producing hydrogen in water, and creating 2D and 3D alumina structures [5]. Their special properties make them well-suited for applications in energy and materials science.

#### 1.5.6. Iron-Derived Nanoparticles

Iron nanoparticles (FeNPs) can be used in many areas, such as catalysts, drug delivery, sensors, and energy storage and conversion [5]. They are also studied for use in solar cells, energy harvesting, water purification, and environmental cleanup [6]. FeNPs are also promising as MRI contrast agents to improve the visibility of tissues and organs [6]. Their magnetic properties make them useful in magnetic storage devices, like hard disk drives [6]. Like all nanoparticles, FeNPs can pose health and safety risks, even though they are used for targeted drug delivery, MRI, and water purification [6]. These uses need careful evaluation to ensure they are safe and effective in medical and environmental applications.

### 1.6. Sustainable Nanoparticle Synthesis

Nanoparticles for biomedical use can be produced using plants and microbes through a green method, which is eco-friendly, cost-effective, safe, and biocompatible [28]. Biosynthesis forms nanoparticles when microorganisms or plants take up metal ions and convert them into nanoparticles using their cellular enzymes [28]. Nanoparticles are classified as intracellular or extracellular based on where they are formed. Biomimetic methods produce NPs with higher catalytic activity while minimizing the use of costly and toxic chemicals [28].

#### 1.6.1. Elements of Eco-Friendly Synthesis

##### Plants

Plants are increasingly used to produce nanoparticles in three ways: within the plant, using plant extracts, or using individual plant compounds (phytochemicals) [29]. Many plants absorb metals and convert them into nanoparticles. Plant parts, such as fruit, root, stem, flower, latex, seeds, leaves, and seed coats, are used for nanoparticle synthesis through hot or cold extraction and Soxhlet methods, which are biocompatible, eco-friendly, renewable, and non-toxic [29]. Plants contain compounds like flavones, ketones, proteins, alkaloids, terpenoids, vitamins, aldehydes, saponins, phenolics, polysaccharides, amino acids, and tannins that help reduce metals into nanoparticles [5]. The process involves mixing plant extracts with metal salt solutions of the corresponding metals such as AgNO_3_, HAuCl_4_, PdCl_2_, H_2_PtCl_6_, Cu (NO_3_)_2_⋅3H_2_O, FeCl_3_⋅6H_2_O, Na_2_SeO_3_, and (NiNO_3_)_2_⋅6H_2_O at the appropriate temperature and pH. Ag, Au, Se, Pt, Cu, Fe and Ni are common nanoparticles [28]. Plant extracts of *Piper nigrum*, *Ziziphus Spina-christi* and *Eucalyptus globulus* were utilized for the synthesis of Ag nanoparticles. Au nanoparticles form in *Brassica juncea* and *Medicago sativa* when exposed to aqueous KAuCl_4_ solutions [10].

##### Bacteria

Prokaryotic bacteria are key in producing metal nanoparticles because they can reduce metal ions [9]. Examples of such bacteria include *Escherichia coli*, *Pseudomonas aeruginosa*, *Plectonema boryanum*, *Pseudomonas stutzeri*, *Salmonella typhi*, *Staphylococcus currens*, and *Vibrio cholerae*. They can produce metal nanoparticles both inside and outside their cells [9,30].

##### Fungi

A highly effective method for producing metal or metal oxide nanoparticles is through the biological processes of fungi [30]. These fungi act as biological agents because they contain special enzymes that help form nanoparticles. Compared to fungi, bacteria produce fewer nanoparticles [28]. For example, researchers used the fungus *Alternaria alternata* culture filtrate to produce platinum nanoparticles, which were spherical and triangular in shape, as shown by spectroscopic analyses [31]. Selenium nanoparticles, chitosan, and fungi were also effective together as reducing agents [31]. Fungal cultures are easy to isolate, simpler to process than bacterial cultures, and produce large amounts of extracellular enzymes [5].

##### Yeast

Microorganisms classified as yeasts are unicellular entities [5]. Many researchers have reported producing nanoparticles using yeast [10]. *Fusarium oxysporum* metabolizes silver nitrate into silver oxide, producing well-dispersed nanoparticles [28]. Its nitrate reductase enzyme helps form highly stable silver nanoparticles in solution [10].

##### Algae

Cyanobacteria and green algae, such as *Lyngbya majuscula* and *Spirulina subsalsa*, can be used as cost-effective sources for recovering metals from liquids [31]. These ancient organisms offer many benefits, including serving as sources of bioethanol and fossil fuels and as effective precursors for modern nanoparticle synthesis [31]. The biomolecules present in these organisms can reduce metals to nanometallic forms and stabilize them. Nanoparticle synthesis can be achieved through two primary methodologies. The first method involves extracellular processing, in which cell metabolites are extracted to facilitate nanoparticle purification [28]. This process enables control of pH, temperature, metal concentration, and substrates to adjust nanoparticle size, shape, and aggregation, making production easier and more efficient [28]. The second method involves intracellular production, where ions move across the cell wall. For example, *Ulva intestinalis* changes color from green to purple after 72 h at 20 °C with chloroauric acid, indicating successful gold nanoparticle (Au-NP) synthesis [28].

##### Actinomycetes

Actinomycetes are important for producing metal nanoparticles, both inside and outside their cells [29]. According to Olawande et al. (2024), Actinomycetes are capable of producing stable and uniformly distributed nanoparticles that exhibit significant biocidal properties against a range of pathogens [29]. Species like *Thermoactinomycete Rhodococcus*, *Streptomyces viridogens*, *Nocardia farcinica*, *Thermomonospora*, and *Streptomyces hygroscopicus* efficiently produce gold nanoparticles (Au-NPs), while *Streptomyces* species mainly synthesize copper, silver, manganese, and zinc nanoparticles [9].

##### Enzymes

Silver nanoparticles (AgNPs) were produced using an enzyme-assisted method on solid surfaces [9]. This method is advantageous because enzymes have defined structures and can be easily purified [9]. Bimetallic iron/palladium (Fe/Pd) particles were directly produced by embedding enzymes into polymer multilayer membranes using electrostatic interactions [32]. For the first time, green tea extracts were used to make Fe/Pd bimetallic nanoparticles, acting as both capping and reducing agents [28]. Additionally, gold nanoparticles (AuNPs) modified with a redox enzyme can form a hybrid electrically active material, where the enzyme transfers electrons between the electrode and biocatalyst, making it useful for sensor applications [32].

##### Vitamins

Vitamin B_2_ enables eco-friendly synthesis of silver and palladium nanospheres, nanowires, and nanorods [10]. In this process, Vitamin B_2_ reduces metals to form nanowires and nanorods. This green nanotechnology approach uses natural agents to explore effects on different tumor cells [10]. Ascorbic acid acts as both a reducing and capping agent by interacting with metal ions, while chitosan stabilizes the nanoparticles [10].

### 1.7. Wet Chemical Methods

Wet-chemical methods include two main approaches: making materials directly from chemical solutions (e.g., solution-based epitaxial growth) and modifying already-formed materials (e.g., ion exchange) [33]. In solution epitaxial growth, new materials nucleate on specific regions of existing particles (called seeds) [34]. Ion exchange, especially when it involves swapping positively charged ions (cations) in a material with those from a solution, is a powerful way to create layered structures composed of different materials, known as epitaxial heterostructures [35].

#### 1.7.1. Solution Epitaxial Growth

In solution-based epitaxial growth, “seeds” (tiny starting particles) are always needed to build layered structures using different methods, such as hot-injection or hydrothermal techniques [33]. Normally, it is hard for new particles to form spontaneously in a solution (homogeneous nucleation) because it requires a lot of energy. If seeds are present in the solution, new material forms more easily on them, a process called heterogeneous nucleation [34]. The size, shape, crystal structure, and surface features of these seeds have a big impact on how the new material forms and grows [35].

##### Crystal-Overlayer Growth Modes

As in vapor-based thin-film growth, layered structures (epitaxial heterostructures) form in solution via three main modes. Layer-by-layer growth (Frank–van der Merwe mode), where one thin layer grows smoothly on top of another; Island growth (Volmer–Weber mode), where small clusters or “islands” of material form instead of a smooth layer; layer-plus-island growth (Stranski–Krastanov)—growth begins as a smooth layer and then shifts to forming islands [33].

##### Synthesis Strategies

High-temperature injection approach

The hot-injection method is a very effective way to make uniform, brightly glowing cadmium chalcogenide nanocrystals (NCs) and to build layered structures with different shapes [34]. In this method, a cool chemical solution (at room temperature) is quickly injected into a hot reaction mixture [34]. This sudden change helps separate the starting step (nucleation) from the later growth of the nanocrystals. As a result, the nanocrystals that form are very similar in size, which means they have a narrow size range [36].

Hydrothermal/solvothermal synthesis technique

The hydrothermal or solvothermal method is a common wet-chemical technique that uses water or organic solvents as the reaction medium. The reaction takes place in a sealed metal container (lined with Teflon) that can handle high pressure [33]. Because the reaction happens at temperatures above the boiling point of the liquid, high pressure builds up inside, which helps the reaction go faster and improves the quality (crystallinity) of the nanocrystals (NCs) that are made [33]. One big advantage of this method is that most starting materials can dissolve well under these high-pressure, high-temperature conditions [33]. It is also easy to use, produces a large amount of material, and is low-cost, making it a popular choice for creating layered nanostructures (epitaxial heterostructures) [35].

#### 1.7.2. Ion Exchange

Ion-exchange methods have become increasingly popular recently. Previously, layered structures (epitaxial heterostructures) were made directly from chemical building blocks, which now serve as starting materials for ion exchange [34]. These well-made structures can be easily changed into new ones with different chemical compositions, while still keeping a similar overall structure [35]. Also, by doing only partial ion exchange, it is possible to create certain structures that would be very hard to make using regular chemical methods [35].

##### Cation Exchange

Cation exchange is a process where positively charged ions (cations) in a nanocrystal are replaced with different cations, while the negatively charged part (the anion structure) stays the same [35]. This method is a very effective way to create many different materials and nanostructures [35]. The rapid progress in solution-based epitaxial growth has made many well-structured layered materials available, which can be easily used for cation exchange [33].

##### Anion Exchange

Unlike cation exchange, which usually preserves the nanocrystal’s shape, anion exchange often alters it significantly [33]. This is due to the Kirkendall effect, which occurs at the nanoscale and causes the structure to shift. When nanocrystals go through this kind of transformation, they often become polycrystalline, meaning they are made up of many small crystal parts. However, in some cases, it is still possible to create layered structures (epitaxial heterostructures) using anion exchange [33].

### 1.8. Nano-Carriers for Immobilization

Nanoparticles provide highly effective supports for enzyme immobilization, using their large surface area, low mass transfer resistance, and optimal loading capacity to enhance biocatalyst performance [37]. Notably, when confronting diffusion challenges posed by macromolecular substrates, nanoparticles emerge as prime candidates due to their unique attributes [38]. Enzyme-coated nanoparticles display Brownian motion in aqueous solutions, highlighting their enhanced catalytic performance compared to free enzymes [39]. Magnetic nanoparticles offer an added advantage, facilitating easy separation through external magnetic fields [40]. Studies elucidate that enzyme immobilization onto nanoparticles mitigates protein unfolding, enhancing stability and overall performance [41]. Numerous studies have investigated various nanoparticles—metal, metal oxide, magnetic, porous, and polymeric—as supports for enzyme immobilization [42]. Enzymes like lysozyme, glucose oxidase, aminopeptidase, and alcohol dehydrogenase have been studied for immobilization on gold (Au) and silver (Ag) nanoparticles, either as isolated enzymes or within whole cells [43]. Notably, Ghulam Kadir [44]. immobilized *S. Carlsberg* and *Candida antarctica* lipase B (CALB) on fumed silica nanoparticles, achieving high catalytic activity in non-aqueous environments. Additionally, Adnan et al. harnessed acetylcholinesterase immobilization onto iron oxide/silica-based magnetic glasses for paraoxon sensing, achieving highly sensitive detection of organophosphate pesticides [42]. Further advances include Adnan, et al. [42] who immobilized acetylcholinesterase on nickel nanoparticles, enabling sensitive detection of organophosphate pesticides. Their application of magnetic polymeric substrates for α-amylase immobilization showcased increased substrate affinity and sustained specific activity over multiple reuses [45]. Notably, cellulase immobilization on magnetic nanoparticles yielded slightly reduced activity compared to free enzymes, albeit exhibiting heightened activity at elevated temperatures [46]. Utilizing amino-functionalized silica-coated magnetic nanoparticles, Ahnan et al. successfully immobilized trypsin, enhancing proteome analysis through pressure-assisted digestion [42]. Innovations persist with the construction of glucose biosensors, where amino-functionalized Fe_3_O_4_@SiO_2_ nanoparticles, covalently attached to ferrocene monocarboxylic acid, it enabled rapid glucose detection, reaching 95% of the steady-state current within 10 s [38]. Recent laboratory studies have focused on immobilizing enzymes such as peroxidase, cellulase, trypsin, and α-amylase on titanium dioxide (TiO_2_) nanoparticles [47]. These immobilized enzymes show increased activity and improved thermal stability at high temperatures, retaining the benefits of micron-sized particle supports [48]. Nanoparticle-based enzyme immobilization represents a burgeoning field, offering a versatile platform for enhancing enzymatic performance across diverse applications.

#### 1.8.1. Electrostatic Adsorption

Among the myriad techniques employed, electrostatic adsorption stands out as the foremost method for linking nanoparticles and proteins [11]. It is widely used as an electron-dense marker in histology, with nanoparticle–protein interactions easily adjusted by pH or by controlling the medium’s ionic strength [48].

#### 1.8.2. Covalent Binding to Functionalized Nanoparticles

Another key method for nanoparticle–protein conjugation is covalent linking of proteins to nanoparticle ligands, optimized by precise control of surface chemistry [5]. This advancement allows for the introduction of a diverse array of organic functional groups onto the nanoparticle surface under mild conditions [9]. A common labeling method uses covalent binding of primary amines to sulfo-NHS esters or carboxyl (R–COOH) groups [9]. Nanoparticles with NHS esters form covalent bonds with lysine’s primary amines, while maleimide-coated nanoparticles react with cysteine thiol groups in proteins [9]. Oxide nanoparticles, like titanium, iron, copper, silver, and gold oxides, can be easily modified via silanization to introduce amino groups on their surfaces [32]. These amino groups act as adsorbents and as coupling sites for attaching proteins, expanding the versatility and utility of nanoparticle-protein conjugation methodologies [32].

#### 1.8.3. Affinity-Based Protein Coupling

Targeted labeling strategies provide an effective approach for conjugating proteins to nanoparticles [32]. For example, nanoparticles coated with streptavidin can bind to proteins that contain biotin. Similarly, nanoparticles coated with antibodies can specifically bind to certain proteins [49]. This precise and selective affinity-based approach underscores the versatility and potential of nanoparticle-protein conjugation techniques in molecular targeting and bioanalytical applications [31]. When nanoparticles are used as biosensors, especially in Förster Resonance Energy Transfer (FRET) or electron-transfer applications, direct conjugation to protein functional groups without intermediate linkers is often preferred [32]. For gold (Au) and silver (Ag) nanoparticles, this can be achieved via Au–thiol or Ag–thiol chemistry, where a protein containing a cysteine residue forms a covalent bond with the nanoparticles [32]. This conjugation involves incubating the protein with the nanoparticles, as the formation of Au–S or Ag–S bonds is energetically favorable [32]. Similarly, for sulfur-containing nanoparticles such as ZnS or CdSe, cysteine can directly form disulfide bonds with surface sulfur atoms [9]. Additionally, His-tags can promote direct binding due to their natural affinity for metal atoms such as zinc (Zn), nickel (Ni), copper (Cu), cobalt (Co), iron (Fe), and manganese (Mn) [9]. These direct chemical interactions offer a robust and efficient means of achieving nanoparticle-protein conjugation, particularly pertinent in biosensing applications where precise molecular interactions are paramount [9]. As carriers decrease in size towards the nanoscale, a cascade of novel implications emerges, predominantly favoring the utilization of nanoscale materials [5]. However, despite the myriad advantages, it is imperative to acknowledge the inherent challenges associated with the employment of nanoparticles for enzyme immobilization, considering overarching perspectives [5]. Recent studies highlight a wide range of nanomaterials available for enzyme immobilization, including carbon nanotubes (CNTs), magnetic nanoparticles, mesoporous materials, nanofibers, nanocomposites, nanorods, sol–gel matrices with nanoscale particles, and single-enzyme nanoparticles [9]. Yet, a primary hurdle to widespread application lies in the elevated cost and complexity of preparing these support systems [9]. Addressing this challenge necessitates the development of strategies or protocols for nanoparticle synthesis characterized by low cost, eco-friendliness, and scalability for large-scale production [9]. By establishing efficient and sustainable synthesis methodologies, the potential of nanomaterials for enzyme immobilization can be fully harnessed, ushering in a new era of advanced biocatalytic applications [9]. Building on the advantages of NP-based enzyme immobilization (e.g., enhanced catalytic stability, facile separation via magnetic fields), the following section details their practical applications across industrial sectors.

### 1.9. Targeted Applications of Immobilized Enzymes

Immobilized enzymes are employed across various industrial sectors, including laboratory-scale organic synthesis, analytical techniques, and medical applications [47]. The versatility of enzymes extends beyond catalysis in aqueous solutions to encompass reactions within inorganic media, thereby enabling immobilized enzymes to catalyze organic synthesis as well [50]. Adnan et al. have extensively documented various applications for immobilized enzymes, including but not limited to high-fructose corn syrup production, pectin hydrolysis, debittering of fruit juices, interesterification of food fats and oils, biodiesel production, and carbon dioxide capture [42]. Notably, the applicability of immobilized enzymes transcends conventional scales, extending seamlessly into the realm of nanotechnology [51].

#### 1.9.1. Stability of Different Types of Nanoparticles in Aggressive Conditions

Recent research from 2024 to 2025 focuses on advanced surface engineering and functionalization strategies to achieve robust, or “aggressive,” nanoparticle stability [14]. Although nanoparticles are naturally prone to instability, innovations in surface chemistry and materials science now allow them to maintain uniform dispersion and resist aggregation, degradation, or loss of function in challenging environments [16]. These harsh conditions can include high temperatures, extreme pH values, high ionic strength solutions, or complex biological matrices. The stability of nanoparticles under such conditions depends on factors such as their core material, size, shape, and most importantly, their surface functionalization, which can be tailored to enhance resilience for specific applications [16].

##### Surface Modification

Applying specialized coatings such as polymers, surfactants, biosurfactants, or organic materials is a key strategy to prevent nanoparticle aggregation [17]. These coatings stabilize particles by providing steric hindrance or electrostatic repulsion, keeping them evenly dispersed in a solution [17].

##### Controlling Environmental Factors

Controlling parameters such as pH and ionic strength helps regulate the surface charge (zeta potential) of nanoparticles, which is essential for maintaining their stable dispersion and preventing aggregation [18].

##### Optimized Synthesis and Preparation

Techniques such as in situ synthesis or carefully controlled two-step methods, combined with optimized sonication, help achieve uniform initial nanoparticle dispersion and effectively break up clusters [18].

##### Different Types of Nanoparticles Alongside Their Stabilities in Aggressive Conditions

(a) Metal Oxide Nanoparticles (TiO_2_, ZnO, CeO_2_, Fe_3_O_4_, CuO)
  (i) Inherent Properties & Stability
    These nanoparticles are prone to agglomeration in dispersion media because of their high surface energy and unsaturated bonds [19]. Their stability is strongly influenced by environmental factors such as pH and the presence of natural organic matter (NOM) [19].
  (ii) Performance in Aggressive Conditions (with proper modification)
    Adsorption of natural organic matter (NOM) can greatly reduce nanoparticle aggregation in natural water. Additionally, surface modification using silane coupling agents or polymers such as PEG, PVA, or PAA provides long-term colloidal stability in biological media and across a broad pH range (2–12) [2].
(b) Polymer/Organic Nanoparticles
  (i) Inherent Properties & Stability
    They generally exhibit high intrinsic biocompatibility and allow for controlled, adjustable degradation rates [20].
  (ii) Performance in Aggressive Conditions (with proper modification)
    These nanoparticles are engineered for stability in physiological conditions, resisting aggregation and immune recognition. Their thermal and chemical stability can be tailored by adjusting polymer chain length and cross-linking [20].
(c) Gold (Au) & Silver (Ag) Nanoparticles
  (i) Inherent Properties & Stability
    These nanoparticles have high intrinsic stability and resist oxidation and corrosion. However, they can aggregate in solutions with high ionic strength if not adequately stabilized [52].
  (ii) Performance in Aggressive Conditions (with proper modification)
    Nanoparticles can remain highly stable in strong salt solutions (e.g., 400 mM NaCl) and various biological media when coated with specific ligands such as anthocyanins or proteins [53]. Surface sulfidation can further reduce ion release and enhance stability under environmental conditions [52].
(d) Carbon Nanotubes (CNTs)
  (i) Inherent Properties & Stability
    These nanoparticles exhibit high thermal conductivity but tend to aggregate, making uniform dispersion difficult due to strong van der Waals forces [52].
  (ii) Performance in Aggressive Conditions (with proper modification)
    These nanoparticles can remain highly stable for over 100 days in specific base fluids, such as water/ethylene glycol mixtures, when optimized surfactants and sonication are used [1]. Their stability strongly depends on the preparation method and the type of surfactant, with cationic or non-ionic surfactants generally performing better than ionic ones [1]. Table 1 shows nanoparticle-bound enzymes and their biotechnological uses

## 2. Overcoming Multi-Drug Resistance

Carbon nanomaterials, particularly carbon nanotubes (CNTs), hold significant promise for biological applications. CNTs exhibit endohedral filling characteristics, encapsulating small molecules within their 2–10 nm diameter [9]. These nanoparticles can be heterogeneously surface-functionalized and cytochemically stained, making them suitable for biorecognition and drug delivery applications [9]. However, the biocompatibility of carbon nanotubes (CNTs) and the difficulty of surface functionalization remain major challenges, limiting their practical applications [32]. Recent studies have explored carbonized polypyrrole nanoparticles (CPyNs) as alternatives [32]. Nanoparticles with controlled diameters exhibit favorable textural properties and, owing to their porosity, magnetic characteristics, and biocompatibility, hold potential as imaging probes and drug delivery carriers [59]. Using oil/water emulsion templating, CPyNs of three distinct sizes; 55, 76, and 99 nm were successfully synthesized [59]. Their microporous structure permits loading of guest molecules through phase separation, while their magnetic properties enable selective separation and targeted delivery [32]. In vitro studies show that these nanoparticles provide sustained drug release, with smaller sizes and amine surface modifications further enhancing this effect [9]. The combination of microporosity, monodispersity, magnetic properties, and biocompatibility makes CPyNs suitable for diverse applications in biomaterials science, including bioimaging and magnetically guided drug delivery [32].

### 2.1. Enhanced Drug Stability and Bioavailability

**Figure 2:**
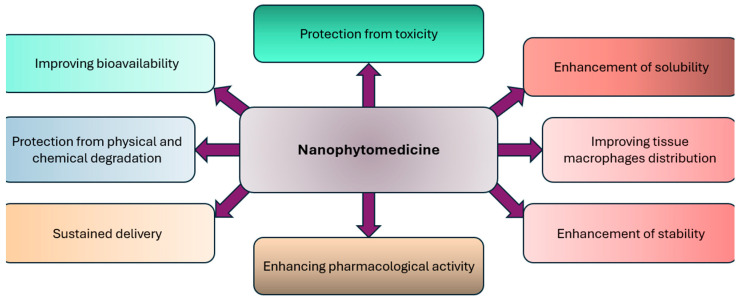
Uses of nanotechnology-based phytomedicine formulations to enhance therapeutic effect [60].

To meet therapeutic goals, a drug must deliver its active ingredient effectively. Through careful design, the absorption rate and extent (bioavailability) of drugs can be adjusted to match therapeutic needs, ranging from fast and complete absorption to slow and sustained release [32]. Phytomedicine, an important part of modern therapy, faces delivery challenges due to poor water solubility, low absorption, limited bioavailability, instability, and extensive first-pass metabolism [9]. The effectiveness of drugs from plant, animal, marine, or synthetic sources depends on their ability to reach the target site in the body at sufficient levels to produce the desired effect [9]. Phytomedicine has long played a key role in drug development, with nearly half of today’s useful drugs derived from natural sources. Its growing use is linked to its strong therapeutic effects and generally fewer side effects compared to conventional medicines [9]. Research on the phytochemical and pharmacological properties of phytomedicines has confirmed their potential. However, despite strong in vitro results, there in vivo effectiveness is often limited by poor water solubility, high lipophilicity, and unsuitable molecular size, resulting in low absorption and poor systemic availability [9]. Understanding the biopharmaceutical and pharmacokinetic properties of phytomedicine is key to creating effective dosage regimens. Nanotechnology, which has the potential to transform medicine and pharmaceuticals, provides many new materials and approaches [32]. Nanotechnology is increasingly benefiting many areas of medicine. Its application in treatment, diagnosis, monitoring, and biological system management, known as nanomedicine, is rapidly growing. In herbal formulation research, nano-based systems offer key advantages for phytomedicine, including improved solubility and bioavailability, reduced toxicity, enhanced activity, greater stability, targeted tissue distribution, sustained release, and protection from degradation [32]. Thus, nano-phytomedicine shows promise for improving the effectiveness of herbal drugs and overcoming their challenges. Applications of nanotechnology-based phytomedicine formulations to enhance therapeutic effect are shown in Figure 2. Table 2 shows dye breakdown by bacterial-derived nanoparticles

### 2.2. Emerging Therapeutic Strategies

#### 2.2.1. Nanoparticle-Mediated mRNA Delivery

Among the emerging therapeutic strategies, RNA-based treatments, especially messenger RNA (mRNA) therapy, show remarkable potential for targeted protein production and gene-based disease intervention [52]. Synthetic mRNA enables rapid and efficient protein synthesis within cells, offering several advantages over traditional protein- or DNA-based therapies [14]. These include controlled protein expression, the absence of insertional mutagenesis risk, and the ability to function without entering the cell nucleus [52]. Moreover, chemical modifications to mRNA can improve translation efficiency while reducing immune-related side effects. A major limitation of mRNA therapy is the instability of mRNA, which can be easily degraded before it reaches the target cells [52]. To overcome this, nanocarriers are used to protect mRNA and facilitate its safe and efficient delivery. Ideal carriers should securely encapsulate mRNA, remain stable in the bloodstream, resist enzymatic degradation, and promote targeted cellular uptake and intracellular release [14]. They must also be biocompatible, cost-effective, and suitable for large-scale production. mRNA delivery systems are broadly classified into viral and non-viral vectors, with non-viral systems preferred due to their flexibility, lower immunogenicity, and simpler production [14]. Non-viral carriers employ various biomaterials, including lipids, polymers, peptides, biomimetic membranes, and inorganic or metal-based nanoparticles. Among lipid-based systems, such as liposomes, lipoplexes, cationic nanoemulsions, nanostructured lipid carriers, and lipid nanoparticles (LNPs) [52]. LNPs are the most advanced and clinically relevant. LNPs have revolutionized non-viral mRNA delivery and are being explored for treating neurodegenerative diseases such as Alzheimer’s disease, Parkinson’s disease, multiple sclerosis, and stroke [52]. They effectively encapsulate mRNA, protecting it from degradation and promoting cellular uptake. Importantly, LNPs can deliver mRNA encoding therapeutic proteins, anti-inflammatory cytokines, or neuroprotective agents, offering a promising platform for modifying disease progression and advancing next-generation neurological therapies [14].

#### 2.2.2. Nanoparticles-Enabled CRISPR-Cas9 Gene Editing

CRISPR/Cas9 (Clustered Regularly Interspaced Short Palindromic Repeats–associated protein 9) genome editing has revolutionized modern medicine, particularly in cancer therapy, by enabling the precise modification of disease-related genes [20]. This system uses a single guide RNA (sgRNA) to direct the Cas9 nuclease to a specific DNA sequence, where it introduces a targeted cut that can either disrupt or repair genes. Compared to earlier gene-editing tools such as zinc finger nucleases (ZFNs) and transcription activator-like effector nucleases (TALENs), CRISPR/Cas9 offers greater precision, efficiency, and versatility, making it a powerful tool for therapeutic applications [1]. Since its initial application in mammalian cells in 2013, CRISPR/Cas9 has become a pivotal technology in cancer research, facilitating tumor gene modification, enhancing immunotherapy, disease modeling, and combination treatments with conventional anticancer drugs [22]. Despite these advances, a major obstacle to its clinical application is the safe and efficient delivery of CRISPR/Cas9 components into target cells. Both the Cas9 protein and sgRNA must reach the cell nucleus for effective gene editing, but traditional delivery approaches, such as viral or physical methods, face limitations like low efficiency, immune reactions, limited carrying capacity, and unintended genetic alterations [18]. Nanotechnology presents a promising solution by enabling encapsulation, protection, and targeted delivery of CRISPR/Cas9 components using nanoscale materials with customizable properties [18]. Nanocarriers can be designed to enhance stability, protect the genetic material from degradation, and facilitate controlled release within specific cells or tissues. For cancer therapy, the key goal is to ensure efficient accumulation of CRISPR/Cas9 at the tumor site while minimizing off-target effects. A wide range of nanomaterials—such as polymers, lipids, porous silicon, mesoporous silica, and metal–organic frameworks—have been explored for this purpose [20]. Their tunable features (size, shape, surface charge, and composition) can be optimized to improve cellular uptake, endosomal escape, and nuclear delivery. Additionally, functionalizing nanocarrier surfaces with targeting ligands enhances tumor specificity, improving the precision and safety of gene editing [1]. Overall, nanotechnology-based CRISPR/Cas9 delivery systems represent a significant step toward achieving safe, effective, and targeted cancer gene therapy [21].

#### 2.2.3. Development of Metal–Organic Framework (MOF)-Based Nanoparticles for Antimicrobial Applications

Bacterial infections are now the second leading cause of death worldwide. In 2019 alone, 33 bacterial pathogens were responsible for 7.7 million of the 13.7 million infection-related deaths reported globally [2]. The widespread misuse and overuse of antibiotics have accelerated the development of antibiotic-resistant bacteria, posing a severe global health threat. To address this crisis, researchers have explored several innovative antibacterial strategies, including photodynamic and photothermal therapies, the use of antimicrobial peptides and bacteriophages, and nanotechnology-based approaches utilizing metal nanoparticles [24]. Among these, nanomaterials have gained particular attention for their strong and adaptable antibacterial properties. However, conventional antibacterial nanomaterials such as silver nanoparticles often face issues like particle aggregation and loss of activity. Likewise, single-component photocatalysts such as titanium dioxide (TiO_2_) and zinc oxide (ZnO) rely heavily on light activation, limiting their effectiveness in dark or complex environments [24]. Metal–organic frameworks (MOFs) have emerged as a promising alternative to overcome these limitations. MOFs are crystalline, porous materials composed of metal ions or clusters linked by organic molecules, forming highly ordered three-dimensional networks [25]. They are known for their exceptionally large surface areas, adjustable pore structures, and versatile chemical tunability. By varying metal centers, organic ligands, and synthesis conditions, MOFs can be engineered to exhibit specific structural and functional properties. As a result of these unique characteristics, MOFs have found growing applications in antibacterial fields [24]. They are being developed as antimicrobial coatings for medical devices to prevent biofilm formation by drug-resistant bacteria, as active materials in food packaging to inhibit microbial growth and extend shelf life, and as ingredients in oral care products to control dental pathogens and plaque formation. Strengthening research on MOFs in antibacterial applications is therefore essential for advancing next-generation antimicrobial technologies [24].

## 3. Different Nanotechnology Strategies to Improve the Bioavailability and Bioactivity of Phytomedicine

Phytomedicines are gaining popularity for their efficacy in treating various diseases with reduced toxicity [11]. However, they face limitations. Nanotechnology offers solutions by reducing phytomedicine size to the nanoscale, enhancing aqueous solubility and membrane permeability [11]. Novel drug delivery systems, such as liposomes, nanospheres, and phytosomes, improve herbal drug delivery by enhancing solubility, stability, pharmacological activity, tissue distribution, sustained release, and protection from toxicity and degradation [80]. Nanotechnology facilitates (1) enhanced delivery of poorly water-soluble phytomedicine; (2) Site-specific delivery to cells or tissues; (3) Transport across epithelial and endothelial barriers via transcytosis; (4) Intracellular delivery of large phytomedicinal macromolecules; (5) combination therapy; (6) Visualization of drug delivery sites through imaging techniques [11].

### Nano-Carriers for Phytomedicine

The main goals of nanoparticle design for drug delivery are to control particle size, surface properties, and drug release, ensuring site-specific action at the optimal therapeutic rate and dose [11]. Nanoparticles provide multiple advantages for delivering phytomedicines: 1. Easily adjustable particle size and surface features for passive or active targeting. 2. Controlled release and degradation through matrix components, allowing high drug loading without chemical modification. 3. Surface properties facilitating site-specific delivery via target ligand attachment [81]. Nano-phytomedicine can be formulated using methods such as high-pressure homogenization, complex coacervation, co-precipitation, salting out, nanoprecipitation, solvent emulsification-diffusion, supercritical fluids, and self-assembly [11]. These methods can generate various nano-phytopharmaceuticals, including polymeric nanoparticles, solid lipid nanoparticles, magnetic nanoparticles, metal and inorganic nanoparticles, quantum dots, polymeric micelles, phospholipid micelles, colloidal liposomes, and dendrimers [11]. Greater attention should be directed toward dendrimers as emerging nanocarriers in phytomedicine. These highly branched, monodisperse macromolecules possess well-defined architectures, tunable surface functionalities, and exceptional loading capacity, making them attractive for the delivery of plant-derived bioactive compounds [1]. Their multivalent surfaces can be engineered to enhance solubility, stability, and targeted delivery of phytochemicals, thereby improving therapeutic efficacy [21].

Dendrimers have been extensively explored in diagnostic applications, where their structural precision and ability to conjugate imaging agents offer significant advantages [22]. However, their broader translation into therapeutic contexts remains constrained by safety considerations. Interactions between dendrimers and cellular membranes can induce structural perturbations in the lipid bilayer, potentially compromising membrane integrity [22]. Such disruptions are often attributed to the high surface charge density and nanoscale size of dendrimers, which facilitate strong electrostatic interactions with membrane phospholipids [18].

Consequently, while dendrimers hold considerable promise as nanocarriers for phytomedicine, their clinical applicability necessitates careful design optimization [1]. Strategies such as surface modification, charge neutralization, and biocompatible functionalization are essential to mitigate cytotoxic effects and ensure safe integration with biological systems [20]. Numerous studies have explored different nanocarriers for phytomedicine delivery.

## 4. Nanocatalysts in Environmental Biotechnology

**Figure 3:**
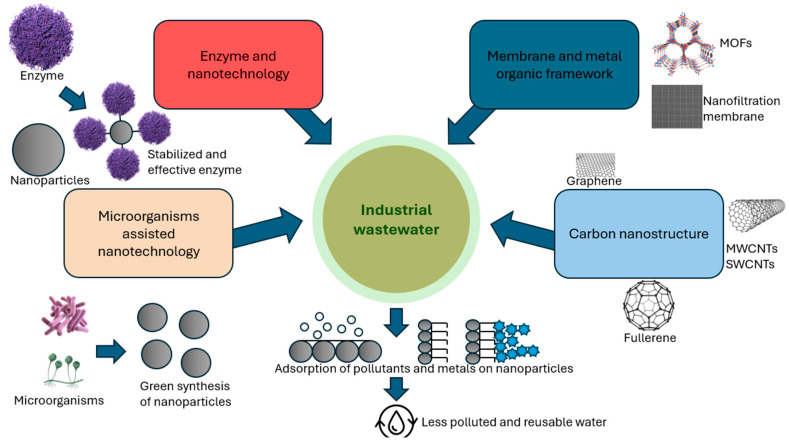
Treatment of industrial wastewater using nanocatalysts.

As the world’s population grows and industries become more advanced, the waste they produce is disrupting our environment [49]. Climate change and pollution are now major global concerns, as they threaten our health and can lead to serious diseases. To protect our planet, it is essential to break down these harmful pollutants as soon as possible [49]. However, certain pollutants are highly resistant to degradation and persist in the environment for long periods, where nanobiotechnology can provide an effective solution [82]. Nanoparticles are widely present in the natural environment. They originate from sources such as sea spray, volcanic eruptions, forest fires, desert dust, and hydrocarbons released by trees (e.g., terpenes that create the blue haze seen in forests) [83]. Some naturally occurring nanoparticles can be harmful to human health. Human activities also generate nanoparticles, though typically in much smaller amounts than natural sources [83]. Major anthropogenic contributors include industrial operations, vehicle exhaust, power plants, jet engines, brake wear, tire abrasion, and wastewater discharges [23]. Common particles released include soot, cerium oxide, metallic dust, calcium carbonate, and silica [16]. Understanding how nanoparticles interact with the environment is an important research focus [16]. Concerns include their ability to transport chemical pollutants through water systems and their potential to disrupt natural or industrial microbial communities, such as those involved in wastewater treatment. At the same time, nanoparticles can provide environmental benefits [19]. They may enhance natural processes and are being explored for applications such as pollutant removal and interactions with organisms ranging from microbes and fungi to plants and animals. Studying these interactions is crucial for advancing environmental science and understanding ecosystems, as soils naturally contain diverse nanoparticles that play key biological roles. By using tiny materials called nanomaterials, scientists can transform dangerous pollutants into harmless substances or speed up the process of breaking them down [82]. Nanomaterials are highly effective due to their large surface area, abundant active sites, and functional groups that readily interact with pollutants. These special properties allow nanomaterials to form strong bonds with different types of pollutants, making it easier to remove them from water and other surroundings [83]. Because of this, nanocatalysts and nanomaterials are widely used for environmental cleanup [84]. Different nanomaterials—such as inorganic, carbon-based, and polymer-based types—can remove pollutants from the environment, enabling effective purification of harmful substances like heavy metals, dyes, pesticides, and toxic chemicals [84]. Processes such as adsorption (trapping pollutants on a surface) and photocatalytic reduction (using light to break down pollutants) are commonly used to clean up contaminated areas. By applying nanobiotechnology, we can significantly reduce pollution and create a healthier, safer environment for the future [82]. Remediation of industrial wastewater using nanocatalysts is an efficient method for breaking down and removing toxic contaminants, as shown in Figure 3.

### 4.1. Nanocatalysts in Heavy Metal Remediation

**Figure 4:**
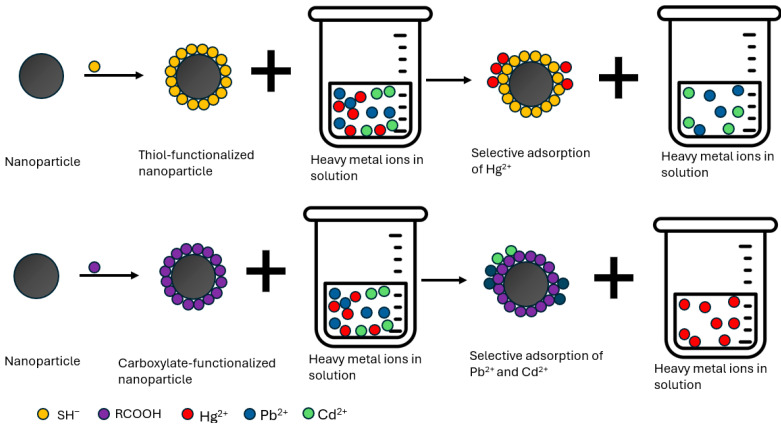
Nanocatalyst in heavy metal remediation.

Heavy metals are major environmental hazards in both soluble and elemental forms, commonly released through industrial activities, poor waste management, and mining. Traditional removal methods like reverse osmosis and chemical precipitation are effective but costly [84]. Therefore, cost-efficient and eco-friendly alternatives are essential. Microbial-derived nanocatalysts offer promising solutions for heavy metal remediation [84]. For instance, palladium nanoparticles synthesized from *Enterococcus faecalis* effectively remove hexavalent chromium from water [31]. Similarly, iron oxide nanoparticles produced by *Aspergillus tubingensis* exhibit high removal rates of metals such as copper, nickel, lead, and zinc from wastewater [82]. This section highlights the potential of microbial-based nanocatalysts for efficient, reusable heavy-metal remediation. Nanocatalyst in heavy metal remediation is shown in Figure 4.

### 4.2. Nanocatalysts in Dye Degradation

The increasing industrial use of dyes, combined with population growth, has caused severe water contamination from untreated waste, creating an urgent need for advanced pollution-control technologies [84]. Nanomaterials are promising for pollution control due to their small size, high aspect ratio, and strong interaction properties. Nanoparticles, in particular, are valuable for catalysis, detection, and environmental remediation, as they can effectively adsorb and degrade a wide range of pollutants from liquids [84]. Bacterial and fungal species have been utilized to synthesize nanoparticles, yielding nanocatalysts that efficiently degrade dyes [85].

## 5. Environmental Remediation

This section provides a clear overview of how nanotechnology is used in environmental restoration, highlighting its potential and challenges. Various human activities contribute to environmental contamination, which poses a significant risk to the balance of ecosystems, public health, and the long-term sustainability of the earth [5]. The contamination of air, water, and soil with harmful substances like toxic metals and organic compounds, as well as emerging pollutants like pharmaceuticals and microplastics, necessitate the development of new remediation strategies that surpass traditional methods [5]. In this context, nanotechnology has emerged as a powerful tool, providing effective solutions for the complex challenges of environmental contamination [5]. Nanotechnology has become a promising field with the potential to significantly change how we approach environmental cleanup. It introduces innovative methods to effectively tackle the wide range of pollution issues currently facing the earth [82]. Nanomaterials have unique properties, including high surface area, tunable surface chemistry, and enhanced reactivity, which make them highly effective for removing, detecting, and monitoring pollutants [82]. Their ability to efficiently and selectively absorb, degrade, or isolate pollutants gives them a major advantage over traditional cleanup methods. Nanoparticles, nanotubes, nanofibers, and other nanostructures are versatile platforms for developing and implementing customized remediation techniques tailored to specific contaminants and environmental conditions [82]. The following table (Table 3) provides additional examples of environmental pollutants and their corresponding photocatalytic agents, showcasing the versatility and potential of photocatalysis in combating environmental contaminants.

## 6. Application of Nanocatalysts in Biotechnology

**Figure 5:**
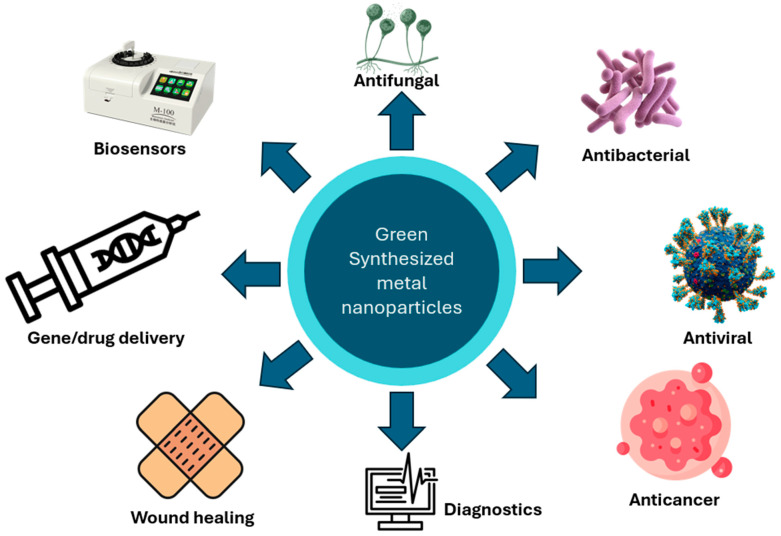
Application of green-synthesized metal nanoparticles.

The term nanobiotechnology refers to the interactions that occur in various systems, particularly in biosensors, where different scientific fields come together [30]. These fields include photonics, chemistry, biology, biophysics, nano-engineering, and nanomedicine. One example of this synergy is the connection between nanoscience and industrial applications, such as the production of sensors used across these disciplines [30]. Nanobiotechnology focuses on understanding how living systems function at the atomic level. To advance this field, scientists manipulate materials at the nanoscale, driving the rapid growth of nanotechnology research [28]. Biotechnology, which examines biological processes including those involving microorganisms plays a crucial role in this collaboration [5]. When combined with nanotechnology, nanobiotechnology contributes significantly to the development of useful tools and instruments for studying life [28]. This field applies nanotechnologies to biological challenges, allowing biologists, physicists, and chemists to approach nanotechnology from their respective perspectives. Their collaborative efforts collectively enhance the understanding and application of nanobiotechnology [10]. Nanotechnology is applied in various industries, such as healthcare, electronics, energy, and environmental management [10]. It provides an effective way to remove contaminants and microorganisms from water via desorption [10]. Moreover, nanotubes have proven effective for delivering drugs, proteins, and cancer-targeting peptides. Various nanoparticles, such as nanowires, nanoshells, and carbon-based nanoparticles, are also used in cancer therapy [10]. The term “green nanotechnology” encompasses the development and application of environmentally benign nanoproducts, which are preferred over conventional chemical methods to foster sustainable development [29]. Figure 5 describes the Application of green-synthesized metal nanoparticles

### 6.1. Impact on Plants

Nanogrowth stimulants promote seed germination effectively due to their small size and high surface area [28]. These nanomaterials can enter seed pores and activate the phytohormones essential for seed growth and germination [28]. For example, applying nano-SiO_2_ and nano-TiO_2_ to soybean plants increases nitrate reductase activity, thereby improving seed germination. The combination of these two nanomaterials was found to be particularly beneficial [28]. In recent studies, biologically synthesized nanoparticles of gold (Au), silver (Ag), titanium (Ti), calcium (Ca), nitrogen (N), and iron (Fe) have gained popularity as nanofertilizers [9]. A study used *Aegle marmelos* plant extract to synthesize iron oxide nanoparticles and evaluate their ability to reduce chromium (Cr) stress in wheat (*Triticum aestivum*). Wheat plants were exposed to 450 ppm of these green-synthesized nanoparticles, and morphophysiological analyses were conducted. Phytochemicals in *Aegle marmelos* acted as natural reducing and capping agents, converting ferric chloride hexahydrate and ferrous chloride solutions into stable iron oxide nanoparticles [9]. Innovative methods addressing plant stress responses have been developed to mitigate the harmful effects of chromium stress on wheat and promote sustainable agricultural practices [10]. Energy-dispersive X-ray (EDX) analysis showed that biologically synthesized iron oxide accounted for 34.91%, whereas chemically synthesized iron oxide accounted for 25.8% [10]. Iron oxide nanorods were successfully synthesized using Withania coagulans extract via a green reduction-precipitation method. These *W. coagulans*-derived nanorods degraded safranin dye 30% more efficiently than chemically synthesized nanorods, as shown by decreased peak intensities at 553 nm and 550 nm under solar light. They also exhibited stronger antibacterial activity against *Staphylococcus aureus* and *Pseudomonas aeruginosa*. Using *W. coagulans* as a bioreducing agent highlights its potential for developing advanced bionanomaterials [10].

### 6.2. Medicines

The nanoscale size of nanoparticles makes them especially valuable in medicine. They can circulate throughout the body, enter cells, and be engineered to target specific tissues [22]. These properties support advanced medical imaging and highly targeted therapies. Nanoparticles improve visualization of organs, tumors, and diseased tissue, and enable treatments such as localized heating (hyperthermia), vascular blockage of tumors, and controlled drug delivery [22]. Magnetic nanoparticles, particularly superparamagnetic iron oxide nanoparticles, enhance magnetic resonance imaging (MRI) and can replace radioactive tracers such as technetium for tracking cancer spread through lymph nodes [1]. Under an alternating magnetic field, these particles can generate heat to destroy tumors. Nanoparticles can also enhance imaging techniques such as fluorescence, PET, and ultrasound when designed to bind selectively to diseased cells [21]. Targeted drug delivery systems use nanoparticles, such as liposomes, nanocapsules, or porous nanosponges, to deliver therapeutic agents directly to disease sites and release them gradually. Inhalable nanoparticles are being explored for delivering drugs to the brain, offering potential treatments for neurological diseases like Parkinson’s, Alzheimer’s, and multiple sclerosis [21]. Nanoparticles and nanofibers also play a key role in tissue engineering. Biocompatible scaffold materials, such as calcium hydroxyapatite nanoparticles combined with collagen, show promise for bone and tissue regeneration [1]. Nanotechnology has also improved consumer health products. For example, the nanoparticle-based sunscreen Optisol replaced traditional zinc oxide or titanium dioxide particles with titanium dioxide nanoparticles doped with manganese, reducing harmful free radical production and improving safety. Advances in lipid nanoparticles (LNPs), pioneered by Pieter Cullis in the 1980s–1990s, transformed drug delivery by protecting therapeutic molecules from degradation [1]. This technology was crucial in developing mRNA vaccines, including the Pfizer-BioNTech COVID-19 vaccine, and continues to support targeted cancer therapy [1]. Green-synthesized nanoparticles (NPs) are valuable in medicine, therapeutics, and in vitro diagnostics. Nanomedicines can bind to biomolecules, thereby reducing tissue inflammation and oxidative stress [5]. They also inhibit cell proliferation and act as effective anticancer agents [5]. Coating silver nanoparticles (Ag NPs) reduces their toxicity and prolongs their biological half-life, enabling targeted destruction of cancer cells [29]. Gold nanoparticles can fight cancer by inducing oxidative stress. They absorb light and convert it into heat, killing cancer cells [29]. Additionally, HIV-1 can readily bind to nanoparticles via its glycoprotein knobs [10]. This nanoparticle–virus interaction blocks the virus from attaching to host cells, helping prevent and treat HIV infection [10]. Furthermore, NPs are highly beneficial in enhancing the bioavailability and solubility of medications, protecting against toxicity, improving pharmacological activities, and ensuring better distribution [10]. They also help prevent physical and chemical degradation, thus increasing the stability of pharmaceuticals within the body [32].

### 6.3. Antimicrobial Activity

Nanoparticles (NPs) produced through environmentally friendly methods have demonstrated significant antibacterial [28], antifungal [32], and antiparasitic properties [9]. Nanoparticles can fight microbes, with metals like silver, copper, gold, platinum, titanium, and zinc showing strong activity [28]. In addition to synthetic antimicrobial chemicals like benzoic, propionic, and sorbic acids, natural nanoparticles made from materials like chitosan or enzymes such as peroxidase and lysozyme also help inhibit microbial growth [5]. Incorporating silver nanoparticles (AgNPs) into gelatin-based nanocomposite films significantly enhances antimicrobial activity against both Gram-negative and Gram-positive foodborne pathogens [5]. Zinc oxide (ZnO) nanoparticles can be incorporated into polymers such as polypropylene, with smaller particle sizes enhancing their antibacterial activity [29]. Nanoparticles such as TiO_2_, ZnO, WO_3_, MgO, Ag_2_O, CuO, and CaO also exhibit potential antibacterial activity against various microbes [5]. In vitro studies show that metal nanoparticles can inhibit bacterial species such as *Escherichia coli*, *Staphylococcus aureus*, *Bacillus subtilis*, and *Pseudomonas aeruginosa* [5]. Bimetallic nanocomposites of silver combined with nickel or cobalt were synthesized using *W. coagulans* extract. The Ag@Co nanocomposite showed strong antibacterial activity against the Gram-positive *Staphylococcus aureus* with 48.9% and 32.1% inhibition, while the Ag@Ni nanocomposite was effective against the Gram-negative *E. coli*, producing 33.1% and 25.7% inhibition zones [5]. Clusters of zinc oxide nanoflowers measuring 30 nm, synthesized through a chemical reduction-precipitation process, showed an average size of 25 nm when using *W. coagulans* fruit extract [10]. Biologically synthesized ZnO nanoflowers exhibited enhanced bioactivity, inhibiting *S. aureus* by 78–88% and *P. aeruginosa* by 85–94% [10]. Chemically synthesized ZnO nanoflowers showed antifungal activity against *Candida albicans* and *Aspergillus niger*, with inhibition rates of 78% and 80%, respectively [10]. *W. coagulans* fruit extract acted as both a reducing and capping agent in the green synthesis of silver and zinc oxide nanoparticles. These nanoparticles possess unique properties and are designed for use against bacterial and fungal pathogens of honey bees (*Apis mellifera*) [5]. The targeted pathogens include *Paenibacillus larvae*, *Melissococcus plutonius*, and *Ascosphaera apis* [29]. Notably, silver nanoparticles and ZnO inhibited growth by 76% and 74%, respectively [29]. ZnO nanoparticles were synthesized using *Fagonia cretica* plant extract. Phytochemical analysis of the extract revealed compounds with antibacterial activity against *Staphylococcus aureus* and *Escherichia coli* [5].

### 6.4. Free Radical Scavenging Activity

Free radicals are highly reactive molecules produced in the body that can initiate chain reactions. This reaction can lead to damage or death of cells [5]. Antioxidants play a crucial role in this context by binding to free radicals, thereby preventing harmful chain reactions and converting these radicals into harmless substances. They help reduce oxidative stress and are used in managing neurodegenerative disorders, aging, cardiovascular diseases, and cancers linked to free radicals [9]. Using *W. coagulans* fruit extract with a chemical reduction-precipitation method, ZnO nanoflowers averaging 30 nm in size were successfully synthesized. At 50 mg/mL, chemically and biologically synthesized ZnO nanoflowers showed antioxidant activities of 56.5% and 67.8%, respectively. Notably, biologically synthesized ZnO nanoflowers, which are less toxic than chemically produced ones, exhibit significant antioxidant activity [9].

### 6.5. Water Remediation

Water from natural sources can be unsafe for human consumption due to organic pollutants (e.g., dyes, pesticides, surfactants), inorganic substances (e.g., fluoride, arsenic, copper, mercury), microbial agents (e.g., algae, bacteria, viruses), and radiological contaminants (e.g., cesium, plutonium, uranium) [9]. Aquatic ecosystem health has declined due to increased wastewater discharge from industrialization and excessive chemical use [9]. Studies have shown that biogenic silver produced by *Lactobacillus fermentum* can remove viruses from drinking water [28]. In these eco-friendly approaches, organisms, their by-products, or nanoparticles are used to remove toxic substances and treat pollutants [28]. Green nanomaterials effectively treat surface water, groundwater, and wastewater contaminated with harmful metals, organic and inorganic compounds, and microorganisms [28]. Self-cleaning nanoscale coatings could potentially replace many conventional cleaning agents. Their growing applications in heavy metal soil remediation and water disinfection highlight their considerable potential [28].

### 6.6. Fuel/Cell

Nanoparticles (NPs) are considered superior materials due to their large surface area, photocatalytic and catalytic properties, optical characteristics, and wide applications in energy production via electrochemical water splitting and photoelectrochemical processes [28]. In addition to reduction and water splitting, other promising energy production methods include electrochemical CO_2_ conversion, solar cells, and piezoelectric generators. Nanoparticles are widely applied in micro-wiring for printed circuit boards in the electronics industry and in metal nanoparticle pastes [32]. Furthermore, these inks can include carbon nanotubes, organic nanoparticles, and ceramic nanoparticles [32]. Metals such as nickel, lead, silver, and platinum are used as catalysts in various chemical reactions, while gold nanoparticles catalyze hydrogenation and oxidation reactions owing to their chemical inertness [32].

### 6.7. Agriculture

Nanofertilizers and nanopesticides are increasingly used in agriculture to control pests, pathogens, and weeds [28]. Nanoparticles containing Mo, Cu, Fe, Ni, Mn, and Zn serve as micronutrients in these fertilizers [32]. Research shows that nanomaterials can enhance plant growth by improving seed germination and development. They penetrate seed pores and activate phytohormones essential for growth [28]. Recently, nanopesticides have become prominent for effectively controlling factors that limit crop growth [29]. Over the past fifty years, the application of nanofertilizers has led to substantial increases in agricultural productivity, especially in grain production, thus aiding in the global effort to meet food demands while minimizing environmental harm [5]. A study examined the effects of chemically and biologically synthesized iron oxide nanorods (NRs) on summer maize (*Zea mays*). Using Moringa oleifera alongside bulk FeCl_3_, the research showed that chemically synthesized NRs and FeCl_3_ at concentrations above 25 mg/L caused growth inhibition and impaired the plants’ physiological and antioxidant functions due to toxic accumulation [10]. In contrast, iron from biologically synthesized nanorods increased growth by 26%, total chlorophyll content by 80%, and nitrate content by 6% [10]. Moreover, treatment with biologically synthesized nanorods enhanced the plants’ antioxidant activity by forming complexes with metal ions [10]. In another study, hydroponically grown lettuce (*Lactuca sativa* L.) was exposed to different concentrations of silver ions and nanoparticles for 25 days to assess their effects on growth. Silver nanoparticles (AgNPs) slightly inhibited the physiological and biochemical functions of plants, including antioxidant activity, demonstrating the phytotoxic effects of AgNPs and AgNO_3_ on seedling development [32]. Ag NP concentrations of 25 and 50 ppm significantly altered protein, soluble sugar, and chlorophyll synthesis compared to the control and AgNO_3_ treatments [32]. At 100 ppm, Ag NPs increased the total reducing potential [32]. These findings suggest that mild stress may enhance plant resilience to pathogens and aid in disease control.

### 6.8. Biology

Nanoparticles (NPs) are used in nanobiotechnology for fluorescent labeling, gene delivery, pathogen detection, protein identification, and DNA analysis. Recently, interest in nanotechnology-based therapeutics has grown significantly [28]. For example, researchers prepared lignin nanoparticles and alginate gel beads, which were used to remove methylene blue from solutions [28]. Most metal nanoparticles are chemically synthesized, which can cause environmental harm, high energy use, and potential health risks. Green synthesis methods have been developed to mitigate these issues [32]. This approach reduces metal ions without harmful chemicals. Green synthesis is preferred over conventional methods because it is more cost-effective, produces less pollution, and is safer for both the environment and human health [32]. Moreover, green synthesis offers an alternative approach for future development, particularly given the environmental challenges and pollution linked to conventional chemical methods.

### 6.9. Biomedical

Iron and iron oxide nanoparticles are weakly magnetic and have diverse medical applications. They help in delivering drugs, repairing tissues, labeling cells, and improving MRI scans [9]. Gold nanoparticles (Au NPs) come in various sizes and exhibit unique optical properties. These properties make them useful in biosensors and cancer treatment [9]. Scientists used a natural plant extract from *Fagonia cretica* to produce zinc oxide nanoparticles (ZnO NPs). Tests showed that these nanoparticles had strong antibacterial effects against two harmful bacteria: *Staphylococcus aureus* and *Escherichia coli* [5]. Another plant, *Withania coagulans*, was used to create highly pure cobalt oxide nanosponges (Co_3_O_4_ NS) using an eco-friendly method. Scientists optimized the process by adjusting the amount of plant extract and the chemical used. The best results were achieved with the least amount of chemical and the most concentrated extract [5]. These cobalt nanosponges have a large, biocompatible surface filled with tiny pores and holes. This unique structure allows them to trap bacteria and kill them by triggering programmed cell death (apoptosis).

### 6.10. Biosensing

Biologically synthesized gold nanoparticles are highly effective for detecting hormones in the urine of pregnant women [5]. Measuring adrenaline in the body is important because this hormone is also used as a medicine to treat allergies, asthma, heart attacks, and during heart surgery [9]. Platinum nanoparticles (Pt NPs) are a new type of biosensor. They are very sensitive and can accurately detect adrenaline in medical tests [9].

### 6.11. Food Industry

Nanoparticles play an important role in the production, preservation, packaging, and delivery of food. In food packaging, they are used in many ways, such as nanosensors, nanoadditives, nanocarriers, anticaking agents, and antibacterial agents [49]. Nanotechnology is used to make nutrient-rich products that help the body absorb nutrients better without changing the food’s taste, color, or texture. These include nano-supplements, powders, nanocochleates, and vitamin sprays with tiny nanodroplets that improve absorption of essential micronutrients [49]. Scientists used Withania coagulans as a natural agent to make zinc oxide (ZnO) and silver (Ag) nanoparticles. These nanoparticles were then tested on Rohu (Labeo rohita), a common carp fish in South Asia [32]. In the experiment, the fish were exposed to nanoparticles for 4 and 15 days to study their effects on blood properties, enzyme activity, and protein levels. The results showed that ZnO nanoparticles worked better than Ag nanoparticles in improving the fish’s survival and cell health. The survival and cellular improvements were recorded at 58%, 69%, and 29% on day 4, and 34%, 51%, and 70% on day 15 [32]. These findings highlight how nanoparticles influence biological systems, both in controlled lab conditions (in vitro) and in living organisms (in vivo).

### 6.12. Electrical

Silver (Ag) and graphene oxide nanoparticles have unique electrical, optical, and physical properties. Scientists studied these 60–100 nanometer nanoparticles using electron and atomic force microscopes [28]. Compared to pure graphene oxide, these nanoparticles showed better optical transparency and higher electrical conductivity. They also had improved permeability and electrical resistance [9].

### 6.13. Catalysis

Metal oxide nanoparticles are widely used as catalysts in chemical reactions, including redox reactions, biosynthesis, green chemistry, and photocatalysis [32]. Iron oxide nanoparticles are important in refining and petrochemical processes. They also help clean the environment by breaking down pollutants [9]. For example, nanoparticles of palladium (Pd NPs) created using soya leaf extracts have been used to break down harmful azo dyes [9]. Scientists also tested iron oxide (Fe_3_O_4_) nanoparticles coated with bio-based materials to remove crystal violet dye, a common pollutant [5]. In these processes, pollutants first attach to the surface of the catalyst, a step called pre-adsorption, which is essential for breaking them down. Many different materials, such as titanium dioxide (TiO_2_), activated carbon, stainless steel, silica, zeolites, and clay have been used to make hybrid photocatalysts for this purpose [5]. Zinc oxide nanofibers (ZnO NFs) were produced using both chemical and biological methods to test their ability to break down pollutants. Researchers used them to remove methylene blue, a toxic industrial dye, under sunlight. The results showed that ZnO NFs effectively degraded the dye, demonstrating their ability to break down harmful organic substances. Scientists used UV-visible spectroscopy to monitor the dye-removal process by measuring changes in light absorption. The study found that up to 90% of methylene blue could be broken down using photocatalytic ZnO NFs, while chemically produced ZnO NFs achieved a 78% breakdown [5]. Researchers also used plant extracts to make bimetallic nanocomposites by combining silver (Ag) with nickel (Ni) and cobalt (Co). These nanocomposites were then tested for their photocatalytic ability to degrade the methyl orange dye. The results showed that Ag@Co degraded 90.2% of the dye, whereas Ag@Ni degraded 82% [10]. Beyond breaking down dyes, these nanocomposites were also highly effective in removing heavy metal contaminants from water. They removed 90% of lead ions at a neutral pH (pH 7) within 45 min. Their strong adsorption capacity and antioxidant properties make them highly promising materials for eliminating harmful pollutants from the environment [10].

### 6.14. Energy Storage

Graphene and carbon nanotube nanoparticles are used in energy storage, biology, and electrolytes because they are highly conductive, highly robust, and heat-resistant [32]. Supercapacitors, which store and release energy quickly, are divided into two main types: electrochemical double-layer capacitors and pseudocapacitors. These supercapacitors provide more power and store more energy than regular batteries, including lithium-ion batteries [9]. In electrochemical double-layer capacitors, energy is stored by the buildup of electric charge on the surface. In pseudocapacitors, energy storage happens through reversible chemical reactions.

### 6.15. Biodiesel Production

As environmental consciousness rises and petroleum reserves decline, biodiesel, comprising monoalkyl esters of fatty acids, garners significant research attention as a renewable fuel alternative [98]. Its use is growing worldwide, especially in countries like Germany, France, Italy, the USA, and Japan [32]. Traditional biodiesel production using homogeneous catalysts faces problems like hard-to-separate products, high water use, and pollution from liquid waste [99]. A shift towards “green” methods employing heterogeneous catalysts is emerging [99]. Despite intensive study of solid-phase catalytic methods, their industrial application remains limited, necessitating further research [100]. Heterogeneous catalytic approaches often encounter mass transfer limitations, time inefficiencies, and low effectiveness [100]. Nanocatalysts, characterized by high specific surface areas and catalytic activities, offer promise in addressing these challenges and have become a focal point of recent investigations [100]. Previous studies showed that the solid base nanocatalyst KF/CaO is effective for biodiesel production, giving yields of over 96% [100]. This porous catalyst, with particles 30–100 nm in size, efficiently converts oils with high acid content into biodiesel. XRD analysis shows a new crystal, KCaF3, forms in the catalyst, improving its activity and stability [100]. The catalyst’s high surface area and large pores allow better contact with the substrate, improving transesterification efficiency [100]. The catalyst’s high surface area and large pores allow better contact with the substrate, improving transesterification efficiency [5].

### 6.16. Nanotoxicology

Nanotoxicology is an emerging field with applications ranging from wound dressings to cancer therapy [10]. Starch-coated silver nanoparticles (Ag-np) were investigated for their toxicity on lung fibroblast and glioblastoma cells [10]. Ag-np induced dose-dependent changes in cell morphology, reduced ATP content, and increased ROS production [101]. DNA damage was seen, causing cells to stop in the G2/M phase, likely because silver nanoparticles (Ag NPs) affected mitochondrial function [101]. The study suggests that silver nanoparticles (Ag NPs) could be used in cancer therapy because cancer cells are more sensitive to them [49]. Nanofilaments, composed of various materials, were synthesized and evaluated for their acute toxicity [9]. Titanate nanotubes and nanowires exhibited dose-dependent cytotoxic effects on lung tumor cells, with enhanced toxicity observed after acid treatment [10]. Structural imperfections resulting from atom diffusion during treatment contributed to the heightened cytotoxicity, cautioning against their manipulation. Gold nanoparticles (GNPs) with different shapes, coated with PEG or CTAB, were studied in microglial cells and neurons. Both cell types took up the GNPs, and microglia showed increased TLR-2 activity [102]. The study shows that the shape and surface coating of gold nanoparticles (GNPs) affect how microglial cells are activated, offering insights into their potential applications in neuroscience [49].

### 6.17. Hydrogen Synthesis for Fuel Cells

Hydrogen fuel cells offer a clean and efficient alternative to fossil fuels, but their widespread adoption hinges on advancements in hydrogen production, storage, and utilization [98]. Water splitting stands out as a renewable, eco-friendly method for producing hydrogen, achievable through various techniques, including electrolysis, photochemistry, and biological processes [98]. While conventional hydride compounds face challenges in onboard hydrogen storage for transportation, nanoscience presents innovative solutions. Fuel cells have great potential to generate electricity for transportation and power grids, cost and performance barriers persist [49]. Aluminum is highly reactive but can become inactive in certain pH ranges. Milling it with water-soluble salts like KCl or NaCl helps prevent this passivation in tap water [99]. This milled aluminum-WIS system rapidly corrodes in tap water, releasing hydrogen and forming solid aluminum hydroxides as by-products, enabling easy recycling [103]. Researchers aim to enhance hydrogen generation rates by improving aluminum particle activity and utilizing the heat released during the reaction [99]. Fresh surfaces are developed through milling with salt, preventing re-oxidation and enabling long-term storage [49]. Immersion of the activated powder in warm water washes away salt covers, initiating high-rate hydrogen release [9]. Reaction efficiency is maximized by reducing water mass to elevate temperature, thereby increasing the hydrogen production rate [104]. Aluminum alloys made by mechanical alloying produce more hydrogen when using this ratio, with the aluminum-bismuth alloy giving the highest average rate [49].

### 6.18. Nanoparticle Applications in Materials

Nanoparticles are increasingly used in catalysis to accelerate chemical reactions, reducing the amount of catalyst required and lowering costs and environmental pollution [52]. Major industrial applications include petroleum refining and automotive catalytic converters. In biological research, nanoparticles, particularly quantum dots, are important tools for fluorescence imaging, enabling cell labeling, biomolecule tracking, medical diagnostics, and advanced imaging techniques [14]. Nanoparticles possess three key interrelated physical properties. First, they are highly mobile in suspension; for example, a 10 nm silica nanoparticle settles extremely slowly in water [15]. Second, they have very large specific surface areas, meaning that a small volume contains an enormous reactive surface area. Third, many nanoparticles display quantum effects that alter their optical, electrical, or magnetic behavior [15]. These characteristics allow nanoparticles to be tailored for a wide variety of compositions and applications. Nanotechnology improves the efficiency, sustainability, and speed of many existing industrial processes by enabling lower material use and greater reactivity. One example is nanoscale zero-valent iron (NZVI), which is used to remediate environmental pollutants such as PCBs. NZVI particles can move through underground rock layers and neutralize contaminants in deep aquifers [52]. Other nanoscale applications include improved coatings, composites, and advanced electronic technologies such as quantum dots, nanowires, and spintronic devices [14]. Many of the unique benefits of nanoparticles arise from their size, motivating efforts to incorporate them into composite materials. A familiar example is the modern rubber tyre, which combines elastomeric rubber with reinforcing fillers like carbon black or silica nanoparticles [14]. However, manufacturing nanocomposites presents challenges, including nanoparticle agglomeration and the loss of nanoscale properties when combined with bulk materials. Despite these challenges, the early 21st century saw rapid growth in the production and use of nanomaterials, especially in nanocomposites. These materials are now widely used in developing new dielectric and magnetic materials, supporting significant advances in engineering and technology [16].

#### 6.18.1. Flame Retardants

Nanoparticles have been investigated as safer alternatives to traditional flame-retardant additives that contain flammable organic halogens or phosphorus, commonly used in plastics and textiles [16]. Research indicates that materials incorporating nanoclays or metal hydroxide nanoparticles can release fewer toxic fumes during major fires compared with materials using conventional additives, suggesting improved fire safety and reduced health risks [17].

#### 6.18.2. Nanoceramics

A major goal in materials science has long been to improve the toughness of ceramics, which are naturally brittle and prone to cracking. By the early 21st century, researchers succeeded in achieving this by incorporating carefully engineered mixtures of nanoparticles into ceramic structures, resulting in stronger and more resilient materials [17]. New types of ceramics are also being developed, including fully ceramic systems and polymer–ceramic nanocomposites. These materials combine the desirable functional properties introduced by nanoparticles such as enhanced electrical, magnetic, and mechanical performance, with the durability and stability of traditional ceramics [18].

#### 6.18.3. Polymers

Similar to the way in which carbon and silica nanoparticles have been used as fillers in rubber to improve the mechanical properties of tyres, such particles and others, including nanoclays, have been incorporated into polymers to improve their strength and impact resistance [18]. In the early 21st century, the increasing use of non-petroleum-based polymers derived from natural sources drove the development of “all-natural” nanocomposite polymers [15]. Such materials incorporate a biopolymer derived from alginate (a carbohydrate found in the cell wall of brown algae), cellulose, or starch; the biopolymer is used in conjunction with a natural nanoclay or a filler derived from crustacean shells. The materials are biodegradable and do not leave behind potentially harmful or unnatural residues [15].

#### 6.18.4. Light Control

In the 1990s, the invention of blue light-emitting diodes (LEDs), which could produce white light more efficiently and at lower cost, transformed modern lighting [15]. To create white light, blue LEDs require coating materials that convert some of the blue light into other colors, such as red, yellow, or green. This can be achieved using very small semiconductor particles that exhibit size-dependent quantum effects [19]. These particles also enabled the development of nanocomposite polymers used in greenhouse coverings. By converting parts of the sunlight spectrum into red and blue wavelengths, which are most beneficial for photosynthesis, these polymers support improved plant growth [19]. In both applications, light conversion is accomplished using submicron inorganic phosphor particles embedded within the polymer material [19].

#### 6.18.5. Batteries and Supercapacitors

Nanocomposite materials can be engineered to have extremely high internal surface areas, allowing them to store electrical charge efficiently in the form of ions or electrons [19]. This property makes them particularly valuable for use in batteries and supercapacitors. Various nanocomposite materials have been developed specifically for electrode applications. In particular, composites based on carbon nanotubes and layered materials like graphene were extensively studied and began appearing in commercial devices in the early 2000s [2].

#### 6.18.6. Food Packaging

Nanoparticles are increasingly used in food packaging to help maintain freshness and prevent microbial contamination [2]. Packaging materials often contain nanoclays or clay-like nanoparticles that reduce moisture penetration and limit gas exchange, thereby preserving food for longer periods. Some nanoparticles, such as nanosilver or nanocopper, also have antimicrobial properties and can be added to packaging to further inhibit bacterial growth [20]. Similarly, antimicrobial nanoparticles are incorporated into paints and coatings, making them particularly useful for surfaces in hospitals, food preparation areas, and other environments where hygiene is critical [20].

## 7. A Comparative and Contrastive Analysis of Green and Chemical Methods for Nanoparticle Synthesis

Research is increasingly focused on producing nanoscale metals through chemical, physical, and eco-friendly (green) methods [32]. Green synthesis is gaining preference over traditional physical and chemical methods because it uses less energy, produces fewer toxic byproducts, requires simpler equipment, and offers milder reaction conditions [32]. Physical methods such as aerosol generation, ultraviolet radiation, and thermal decomposition typically require high temperatures and pressures, whereas green synthesis uses organic, environmentally safe reducing agents [9]. Makhesana et al. (2024) reported that some eco-friendly materials can act as both capping and dispersing agents, helping to lower energy use and eliminate the need for harmful chemicals [9]. Polyphenols and proteins in green materials can serve as reducing agents, converting metal ions into stable forms without the need for chemical reagents [5]. In some cases, metal nanoparticles made using green methods show better quality than those produced chemically. For instance, Havelikar et al., (2024) found that Fe_3_O_4_ nanoparticles synthesized by green methods had sizes between 2–80 nm, which are much smaller than the 87–400 nm sizes obtained from traditional wet chemical methods [105]. A new approach has emerged for producing zinc oxide (ZnO) nanoflowers (NFs) using cost-effective and stable plant-based methods. In one study, the effects of chemically synthesized nanowires (CS NWs) and biosynthesized nanofibers (BS NFs) on soybean plants were analyzed with a gel-free, label-free proteomic method. ZnO at 10 ppm increased both root and hypocotyl length and weight. The findings also revealed that ZnO affected redox balance, protein folding, and hormone metabolism, with CS NWs and BS NFs causing different protein changes [5]. In CS NWs, ZnO did not affect the levels of heat shock protein 70 (HSP70), whereas in BS NFs, ZnO led to an increase in HSP70 [5]. These results suggest that BS NFs ZnO may support soybean growth by improving protein folding, where increased HSP70 and changes in redox metabolism help detoxify hydrogen peroxide. In contrast, CS NWs ZnO reduced protein folding and caused greater oxidative stress in the plants [5]. Conventional fertilization often leads to problems like eutrophication and higher soil acidity. This study also examined how foliar fertilization with Cordia-based silver nanoparticles (AgNPs) affects biomass production, antioxidant activity, and both morphological and anatomical traits in lettuce [32]. Recently, the use of nanopesticides and nanofertilizers in agriculture has grown, with nanoparticles being produced through both chemical and biological methods [32]. Nano-pesticide and nano-fertilizer emulsions have proven more effective than traditional soil-based applications. However, further studies are needed to better understand the key physicochemical properties driving these effects.

## 8. Toxicity of Nanoparticles

Nanoparticles (NPs) released into air, water, and soil from different industries generate nanowaste, which harms living organisms and disturbs ecosystem balance [31]. Exposure to nanoparticles can raise the risk of health issues, including asthma, allergies, diabetes, cancer, and inflammation [5]. Some nanoparticles, like gold (Au) and titanium dioxide (TiO_2_), can negatively affect the reproductive systems of animals [29]. Nanoparticles such as silver (Ag), copper (Cu), zinc oxide (ZnO), and nickel (Ni) can reduce the activity of key enzymes in microorganisms, potentially disrupting the food chain in ecosystems [29]. In plants, excessive nanoparticles can cause toxicity by damaging cell membranes via lipid peroxidation. This damage can harm DNA, reduce photosynthetic pigments, decrease biomass, and lower protein levels in plants [10]. The harmful effects of nanoparticles on plants can be identified through signs like DNA damage in cells or changes in growth and function. Because of their tiny size, nanoparticles can also cross the blood–brain barrier and reach the brain [10]. Because of their unique properties, they can easily penetrate cell layers, tissues, and organs, potentially causing cellular damage. The larger the surface area of a nanoparticle, the more reactive and toxic it can become [10]. Most of the harmful effects of nanoparticles, such as oxidative stress, occur primarily in vital organs, including the central nervous system [5].

### 8.1. Differentiated Analysis of Toxicity Mechanisms Based on NP Properties

#### 8.1.1. Size

The physical and chemical properties of nanoparticles (NPs) play a crucial role in determining their absorption, transport, and accumulation in living tissues, factors that ultimately influence their biological fate and toxicity [104]. Studies have shown that these physicochemical properties significantly affect nano–bio interactions and potential toxicity, underscoring the need to understand them when designing safer metal-based NPs [105]. The toxicity of metal-based NPs is largely determined by their chemical composition, size, shape, and surface chemistry [106]. Among these, particle size is particularly important because it affects how NPs enter cells and distribute within tissues. Due to their nanoscale size, comparable to biological structures like proteins, DNA helices, and cell membranes, NPs can readily penetrate cells and even reach intracellular organelles [107]. Generally, smaller NPs can pass directly through cell membranes by translocation, whereas larger NPs rely on processes such as phagocytosis, micropinocytosis, or nonspecific transport. Smaller NPs are also more permeable and can interact with organelles such as mitochondria, lysosomes, and the nucleus, potentially leading to cellular damage. For example, in MCF-7 breast cancer cells, gold (AuNPs) smaller than 10 nm (specifically 2 and 6 nm) were able to reach the nucleus, while larger NPs (10 and 16 nm) remained confined to the cytoplasm [107]. Similarly, smaller titanium dioxide (TiO_2_) NPs (6 nm) caused greater oxidative stress and DNA damage in zebrafish embryos under light exposure compared to larger ones (12 and 15 nm). Size-dependent biodistribution has also been observed in vivo. For instance, 10 nm Au NPs were distributed across multiple organs, including the blood, liver, spleen, kidney, heart, lungs, and brain, whereas larger particles were limited mainly to the liver and spleen [108]. Likewise, smaller silver (AgNPs) (10 nm) showed broader tissue distribution and induced more severe liver toxicity than larger NPs (40 and 100 nm) [104]. Furthermore, NP size can influence the mechanism of toxicity. In one study, 1.4 nm AuNPs induced necrosis, while slightly smaller 1.2 nm particles triggered apoptosis, suggesting that even small variations in size can alter cellular outcomes [107]. Overall, smaller NPs possess a higher surface area-to-volume ratio, which increases their chemical reactivity and enhances interactions with cellular components, factors that collectively contribute to their greater toxicity potential [105].

#### 8.1.2. Surface Chemistry

Beyond size and shape, the surface chemistry of nanoparticles (NPs) is another key factor influencing their biological behavior and toxicity [104]. One of the most important surface characteristics is surface charge, which affects how NPs interact with biological molecules, cell membranes, and organelles, as well as their overall movement and clearance in the body [104]. The zeta potential (ζ-potential) is commonly used to measure NP surface charge and predict their potential toxicity. Studies have shown that positively charged NPs are generally more toxic than negatively charged ones [104]. This is because cell membranes are negatively charged, leading to stronger electrostatic attraction between them and positively charged NPs. These interactions increase cellular uptake, resulting in greater cell damage [105]. For instance, positively charged gold (Au) NPs were found to be more cytotoxic than negatively charged ones due to their enhanced internalization by cells [107]. Similarly, magnetic nanoparticles such as ferroferric oxide (Fe_3_O_4_), oleic acid-coated Fe_3_O_4_, and carbon-coated Fe showed varying degrees of toxicity in human liver cancer (BEL-7402) cells depending on their surface charge [105]. Higher positive charges led to increased cell cycle arrest and apoptosis, caused by stronger electrostatic attraction and prolonged contact with cell membranes. In biological environments, metal-based NPs can bind with various proteins such as albumin and immunoglobulins to form a protein corona on their surface [107]. This corona changes the NP’s surface characteristics, influencing its pharmacological (how it behaves in the body) and toxicological properties. However, this interaction can also alter the structure and function of bound proteins, potentially disrupting biological processes or triggering immune responses. Depending on the composition, NP-protein coronas can either stimulate or suppress the immune system, affecting overall cytotoxicity [104]. Surface modifications with materials such as polyethylene glycol (PEG), poly (lactic-co-glycolic acid) (PLGA), polylactic acid (PLA), or lipids are commonly used to improve NP stability and biocompatibility [104]. These coatings can change NP biodistribution, clearance, and elimination, all of which influence toxicity. For example, among three types of aluminum oxide (Al_2_O_3_) NPs pristine (p-Al_2_O_3_), hydrophilic (w-Al_2_O_3_), and lipophilic (o-Al_2_O_3_), the lipophilic o-Al_2_O_3_ NPs were the most toxic both in vitro and in vivo, causing greater cell membrane damage and reactive oxygen species (ROS) generation [5]. On the other hand, some surface modifications can reduce NP toxicity and improve tissue specificity. For instance, coating zinc oxide (ZnO) NPs with a silica layer significantly decreased their cytotoxicity in human dermal fibroblast cells. This coating reduced enzyme leakage, ROS formation, and oxidative stress by limiting free radical generation, slowing the release of zinc ions, and minimizing direct contact between the NPs and cell membranes [104]. Overall, the surface chemistry of NPs, especially their charge and surface modifications, plays a central role in determining their biocompatibility, cellular interactions, and toxicity profile [104].

#### 8.1.3. Shape

In addition to size, the shape of nanoparticles (NPs) plays an important role in determining their biological behavior and toxicity [104]. NPs can exist in many shapes, such as spheres, rods, cubes, cylinders, ellipsoids, sheets, or discs, and even when they share the same size and composition, their shape can significantly influence how they interact with biological systems [107]. These effects include differences in biodistribution, cellular uptake, deposition, and clearance from the body. Computational models, such as coarse-grained molecular dynamics (CGMD), have shown that NP shape influences how particles are taken up by cells during endocytosis [105]. Variations in shape affect the local curvature energy of the cell membrane, which, in turn, determines the endocytic pathway and the angle of particle entry. Experimental evidence also shows that non-spherical NPs (such as rods or discs) are often internalized more efficiently and in greater quantities than spherical NPs [105]. Shape also affects circulation and organ accumulation. For example, rod-shaped PEGylated gold (Au) NPs circulate longer in the bloodstream and accumulate more effectively in tumors compared to spherical Au NPs. Similarly, discoidal porous silicon nanovectors accumulated five times more in tumor tissues of breast cancer-bearing mice than spherical particles of similar sizes [109]. Changes in NP shape can also alter surface area, influencing their reactivity and toxicity. For instance, rod-shaped iron oxide (Fe_2_O_3_) NPs caused greater toxicity in mouse macrophage cells (RAW 264.7), as shown by increased lactate dehydrogenase (LDH) release, tumor necrosis factor-α (TNF-α) production, reactive oxygen species (ROS) generation, and cell necrosis compared to spherical Fe_2_O_3_ NPs [109]. Similarly, rod-shaped cerium oxide (CeO_2_) NPs induced higher LDH release and TNF-α production than cubic or octahedral CeO_2_ NPs with the same chemical makeup. However, other studies reported contrasting results: for example, in human liver (HepG2) cells, cube-shaped CeO_2_ NPs were the most toxic, while rod-shaped NPs were the least toxic. Additionally, spherical titanium dioxide (TiO_2_) NPs were found to be five times more lethal to *Escherichia coli* than elongated TiO_2_ particles [104]. These findings suggest that NP shape strongly influences bioaccumulation, cellular interactions, and surface reactivity, all of which contribute to their varying degrees of toxicity [104].

## 9. Challenges and Potential Risks of Nanotechnology Applications

Nanoparticles (NPs) have many applications and offer great potential for large-scale production [32]. Nanotechnology is a promising field that could strongly drive future progress in nanoscience [32], particularly by leveraging waste materials and algae to produce eco-friendly nanomaterials. At present, most nanomaterials are made using organic solvents, which can pose serious risks to reproductive and neurological health during production [29]. The use of high pressure and heat can also create unsafe working conditions. A major concern is the release of volatile vapors and large amounts of carbon dioxide, both of which greatly contribute to greenhouse gas emissions [29]. Other key factors to consider include bioavailability, possible side effects, interactions with cells, and biodegradation [9]. Bringing nanomedicines into clinical use takes significant time and effort because of potential toxicity risks [9]. There is an urgent need to replace the hazardous nanoparticles mentioned earlier, but this requires balancing high reaction yield with environmental sustainability [9].

### 9.1. Environmental and Health Implications

When nanomaterials are released into the environment, their unique properties may cause serious risks to human health and ecosystems, including toxicity, buildup in living organisms, and ecological damage [12]. To reduce these risks, researchers and policymakers need to work together to create clear guidelines for the safe use, handling, and disposal of nanomaterials, helping to limit their environmental impact [12]. Strict workplace safety measures and detailed toxicity testing are essential to protect public health. Developing safer nanomaterials and ensuring responsible disposal are also key to reducing risks and supporting environmental sustainability [9].

### 9.2. Regulatory Challenges

Nanotechnology is advancing so quickly that regulations and standards often lag, which can create unexpected risks [12]. To meet this challenge, regulators should collaborate with scientists and industry to develop flexible rules that keep up with technological advances. Standardization is also important to ensure nanomaterials are used safely, effectively, and consistently, reducing risks while maximizing benefits [9].

### 9.3. Ethical and Social Implications

In nanotechnology, issues of privacy, security, fairness, and the responsible use of advanced technologies must be carefully addressed [12]. Open discussion and public involvement are vital for addressing these concerns. Ethical guidelines and regulations should be created to ensure nanotechnology is transparent, protects individual rights, and provides fair access to its benefits for everyone [9].

### 9.4. Cost and Accessibility

Although nanotechnology provides cost-effective solutions by utilizing minimal material with exceptional properties, the initial expenses associated with research, development, and implementation remain substantial [12]. Making nanotechnology-based innovations affordable and accessible, especially in low-resource settings, remains a major challenge. Overcoming this requires strategic investments, capacity building, and fair distribution systems to connect advanced technologies with underserved communities [32].

### 9.5. Capacity Building and Technology Transfer

Many countries struggle to fully benefit from nanotechnology due to weak infrastructure, limited expertise, and scarce resources [4]. To reduce these gaps, responsible technology transfer is needed to ensure nanotechnology benefits everyone. Approaches such as open access to research, technology-sharing agreements, and fair licensing can help address these challenges. These efforts are vital for making nanotechnology globally accessible, especially in underserved regions [32].

### 9.6. Intellectual Property and Innovation

Intellectual property (IP) rights can create major obstacles to sharing nanotechnology solutions, especially in regions with limited resources [4]. To overcome these challenges, it is important to support open access to research, encourage technology-sharing agreements, and create fair licensing systems. Balancing IP protection with the need for broad accessibility will help expand the use of nanotechnology, ensuring its benefits reach diverse populations and support global development [32].

### 9.7. Public Awareness and Acceptance

Public perception is a key factor in how nanotechnology is adopted and applied. Addressing public concerns, fostering transparency, and engaging communities are essential to building trust and acceptance of nanotechnology-based solutions [6]. Effective communication of scientific findings, particularly through clear and accessible illustrations of recent research, is crucial during the development phase. These efforts help bridge the gap between scientific advancements and societal understanding, ensuring informed public support for nanotechnological innovations [32].

## 10. Conclusions

Humans are continuously exposed to naturally occurring nanoparticles and have, to some extent, adapted to background environmental levels. However, nanoparticles generated through anthropogenic activities such as those present in tobacco smoke and combustion emissions are associated with significant health risks, including respiratory injury and premature mortality. For instance, traditional cooking stoves widely used in developing regions emit high concentrations of fine and ultrafine particulate matter, contributing to increased incidence of respiratory diseases, particularly among women with prolonged exposure.

A key mechanistic concern underlying nanoparticle-induced toxicity is the generation of reactive oxygen species (ROS), a phenomenon not restricted to zinc oxide nanoparticles but broadly observed across diverse nanomaterials, including silver, titanium dioxide, and other metal-based nanoparticles. Excessive ROS production can disrupt cellular redox balance, leading to oxidative stress, lipid peroxidation, protein dysfunction, and DNA damage. While such effects are well documented in vitro and animal models often under high-exposure conditions, their relevance to typical human exposure scenarios remains less clearly defined.

Toxicological studies have yielded variable and sometimes inconclusive findings. Inhalation studies in animal models frequently employ elevated nanoparticle concentrations, demonstrating dose-dependent pulmonary inflammation and genotoxicity. Similarly, ingestion studies suggest that certain nanoparticles, such as titanium dioxide (E171), may alter gut microbiota composition and intestinal homeostasis, with potential implications for disorders such as inflammatory bowel disease. However, the long-term effects of chronic, low-dose exposure in humans are still insufficiently understood.

In response to these uncertainties, stringent occupational safety measures are commonly implemented to limit nanoparticle exposure during manufacturing and handling. Regulatory oversight, coupled with increasing public awareness and advocacy, continues to promote safe use and risk mitigation.

Despite these challenges, nanoparticles remain a cornerstone of innovation in biotechnology and medicine. Their unique physicochemical properties have enabled the emergence of nanomedicine, facilitating targeted drug delivery, enhanced diagnostics, and novel therapeutic strategies. Nevertheless, the full extent of their impact is still evolving. Future research should prioritize the development of cost-effective and sustainable green synthesis approaches, the optimization of surface engineering to reduce toxicity, and the advancement of multifunctional nanoparticle-based theranostic systems for precision medicine.

## Figures and Tables

**Table 1:** Nanoparticle-bound enzymes and their biotechnological uses.

Nanoparticle	Enzyme	Classification	Application	References
Silver nanoparticles	Alpha amylase	Nanomaterial	Starch hydrolysis	[53]
Fe_3_O_4_ nanoparticles	Lipase	Magnetite nanoparticles	Hydrolysis of pNPP	[54]
Con A layered ZnO nanoparticles	Β-Galactosidase	Inorganic metal oxide nanoparticles	Lactose hydrolysis	[55]
Fe_3_O_4_ nanoparticles	Keratinase	Magnetite nanoparticles	Synthesis of keratin	[50]
Chitosan-magnetic nanoparticle	Laccase	organic, polymeric nanoparticles	Bioremediation of environmental pollutants	[43]
Silica-coated iron oxide nanoparticle	Haloalkane dehalogenase	metal oxide nanoparticles	Production of fusion proteins containing dehalogenase sequences	[56]
Gold and silver	*Thermoanaerobacter brockii* alcohol dehydrogenase (TbADH)	inorganic nanoparticles	Alcohol synthesis	[57]
Magnetite silica nanoparticles	Horseradish peroxidase (HRP)	non-porous silica nanoparticles” and “mesoporous silica nanoparticles (MSNs)”	Immunoassays	[51]
Chitosan nanofibers	Lysozyme	biopolymer-based nanoparticles	Antibacterial	[58]
Silica nanoparticles	α-Amylase	non-porous silica nanoparticlesandmesoporous silica nanoparticles (MSNs)	Formulation of detergent for enhancing removal of starch soils	[40]
Nano Fe_3_O_4_ coated on a gold electrode surface	Superoxide dismutase (SOD)	magnetite nanoparticles	Biosensors	[56]
Iron oxide	β-Glucosidase (BGL) from *Aspergillus niger*	metal oxide nanoparticles	Biofuel production	[57]

**Table 2:** Dye breakdown by bacterial-derived nanoparticles.

Dye	Synthesis of Nanoparticle Source	Nanoparticle Used	% Degradation	References
Rhodamine B, Methyl orange, Methylene blue	*Sargassum serratifolium*	Gold and silver	-	[61]
Malachite green	*Gracilaria corticata*	Silver	-	[62]
Rhodamine B	*Turbinaria conoides*	Gold	-	[63]
Malachite green	*Escherichia* sp. *SINT7*	Copper	90.55	[64]
Rhodamine B	*Cladosporium oxysporum AJPO3*	Gold	-	[65]
Acid Brillant Scarlet GR	*Trichoderma* spp.	Gold	94.7	[66]
Methyl orange	*Fusarium oxysporum*	Platinum	-	[67]
Bismarck brown	*Aspergillus niger*	Zinc-oxide	89	[68]
Naphthol Green B	*Pseudoalteromonas* sp. *CF10.13*	Iron-Sulfur	19.46	[69]
Direct blue 71	*Saccharomyces cerevisiae*	Palladium	98	[70]
Congo red	*Pestalotiopsis versicolor*	Silver	91.56	[71]
Methylene blue	*Saccharomyces cerevisiae*	Silver	80	[72]
Malachite green	*Akremonium Kiliense*	Silver	95.4	[73]
Congo red	*Pleurotus sajor caju*	Silver	78	[74]
Methyl orange	*Erwinia herbicola*	Tin (iv) oxide	94	[75]
Amaranth	*Shewanella decolorotionis*	Iron	90.5	[76]
Methyl orange	*Clostrodium* spp.	Palladium	90	[77]
Reactive black 5	*Pseudomonas putida*	Palladium	100	[78]
Malachite green	*Bacillus paralichineformis*	Silver	90	[79]

**Table 3:** Contaminants investigated over the last two decades, and the corresponding nanocatalysts applied.

Pollutant	Nanocatalyst	Year	Enhancement Type	Success Note	References
Procion H-EXL	TiO_2_	2010	-	-	[86]
Phenol compounds	TiO_2_-H_2_O_2_	2007	Coupled systems Ion doping	-	[87]
o-Chlorophenol	Cobalt-doped TiO_2_ thin film	2004	Nano-grained films	-	[82]
Indigo carmrine	Manganese- and lanthanum-doped ZSM-5	2005	Ionic doping	Removed these contaminants from water	[88]
Acid Blue 74	MnO_x_-TiO_2_	2006	Interconnected systems	Removed these contaminants from water	[82]
Copper(II) cyanide	TiO_2_	2004		Eliminated these contaminants from water	[86]
Isopropanol	TiO_2_@SnO_2_, TiO_2_@SnO_2_	2006	Coupled/capped systems		[86]
C.I. Reactive Black 5	ZnO film	2005	Nanocrystalline films		[89]
Direct blue dye (DB53)	Ln doped TiO_2_	2009	Ion doping		[56]
Cyanide anion	TiO_2_-SnO_2,_ V_2_O_5_-SnO_2_	2003	Coupled systems		[90]
Methylene blue	Cds-TiO_2_	2006	Coupled systems	Used visible light, >420 nm inhibited undesirable crystal growth	[47]
Gaseous organic compounds	ZrO_2_-modified TiO_2_-_x_N_x_	2006	Coupled systems	Used visible light, >420 nm inhibited undesirable crystal growth	[79]
Organic azo dyes	TiO_2_-BaFe	2004	Coupled systems	Magnetized nanoparticles	[91]
Mercury vapor spores	SnO_2_-TiO_2,_ TiO_2_-multi-walled-carbon nanotubes	2006	Coupled systems CNTS, coupled systems	Effective against bioterrorism	[92]
CO_2_	TiO_2_	2009		14 nm size yields most methanol and methane	[93]
Orange II	TiO_2_	2007		Peptizer: TTIP 1:10 ratio performs better	[41]
Phenol	TiO_2_	1991		After 120 h, no loss of catalyst	[94]
Phenol	TiO_2_	1993		No photocorrosion	[95]
N_2_	Cr(III) doped TiO_2_	1994	Ion doping	Improved charge separation of e- h^+^ pairs	[96]
Phenol (C_6_H_5_OH)	Cr(III) doped TiO_2_	1994	Ion doping	Improved charge separation of e- h^+^ pairs	[97]
Methyl orange	ZnO-SnO_2_	2010	Coupled systems	Cube morphology and Zn:Sn 2:1 ratio perform better	[97]

## Data Availability

Not applicable.
